# Evidence for temporal relationship between the late Mesozoic multistage Qianlishan granite complex and the Shizhuyuan W–Sn–Mo–Bi deposit, SE China

**DOI:** 10.1038/s41598-021-84902-6

**Published:** 2021-03-12

**Authors:** Yuzhong Liao, Bo Zhao, Dehui Zhang, Leonid V. Danyushevsky, Tonglin Li, Mingqian Wu, Feng Liu

**Affiliations:** 1 Institute of Hydrogeology and Environmental Geology, Chinese Academy of Geological Sciences, Shijiazhuang, 050061 China; 2 Nanhu Laboratory, Research Center for Big Data Technology, Jiaxing, 314000 China; 3 School of Earth Sciences and Resources, China University of Geosciences, Beijing, 100083 China; 4 CODES and Earth Sciences, University of Tasmania, Hobart, Australia; 5 Sichuan Earthquake Administration, Chengdu, 610000 China; 6 Department of Earth and Environmental Sciences, University of Windsor, Windsor, ON N9B 3P4 Canada; 7 Technology Innovation Center for Geothermal & Hot Dry Rock Exploration and Development, Ministry of Natural Resources, Shijiazhuang, China

**Keywords:** Geochemistry, Mineralogy, Petrology

## Abstract

The world-class Shizhuyuan W–Sn–Mo–Bi deposit is spatially related to the Qianlishan granite complex (QGC) in Hunan Province, China. However, the age and classification of the QGC are still debated, and a better understanding of the temporal genetic relationship between the QGC and the Shizhuyuan deposit is essential. Here, we present chemical compositions the intrusive phases of the QGC and the results of detailed zircon U–Pb dating and muscovite Ar–Ar dating of a mineralized greisen vein. Our new zircon laser ablation inductively coupled plasma mass spectrometry U–Pb age data constrain the emplacement of the QGC to 155–151.7 Ma. According to petrological, geochemical and geochronological data and the inferred redox conditions, the QGC can be classified into four phases: P_1_, porphyritic biotite granites; P_2_, porphyritic biotite granites; P_3_, equigranular biotite granite; and P_4_, granite porphyry dikes. All phases, and especially P_1_-P_3_, have elevated concentrations of ore-forming metals and heat-producing elements (U, Th, K; volume heat-producing rate of 5.89–14.03 μWm^−3^), supplying the metal and heat for the metalogic process of the Shizhuyuan deposit. The Ar–Ar muscovite age (154.0 ± 1.6 Ma) of the mineralized greisen vein in the Shizhuyuan deposit is consistent with the emplacement time of the QGC, suggesting their temporal genetic relationship.

## Introduction

Magmatic fractionation and exsolution of a fluid phase from a cooling pluton plays an important role in metal enrichment for intrusion-related deposits^1–3^. Reheating of preexisting semi-solidified plutons triggered by the input of a new magma may lead to the exsolution of fluid and element transport, thus contributing to incremental extraction of metals from the magma and their precipitation in the cupola of plutons^4,5^. Successive magma inputs led to the repeated extraction and precipitation of metals to form ore at the vantage position^2,6^. The lifespan of the magmatic-hydrothermal system triggered by the emplacement of a set of successive plutons controls the timescale of the ore-forming process and thus the metal grades and tonnages of deposits. Therefore, establishing tight temporal ties between magmatism and its associated mineralization is key to understanding the contribution of magma to mineralization. It is generally considered that lifespans of magmatic-hydrothermal systems is less than 10 million years, and even shorter (< 2 Ma) for porphyry deposits^7–12^. Accordingly, a time interval between plutons and the associated mineralization of > 10 Ma is interpreted to indicate that they have no genetic ties.

The development of W, Sn, Pb, Zn, and rare earth element (REE) deposits is genetically associated with voluminous Mesozoic granites^13–20^. The Qianlishan granite complex (QGC) in China is located ~ 16 km southeast of Chenzhou city, Hunan Province. The QGC is centered in the well-known Shizhuyuan polymetallic ore zone (W–Sn–Mo–Bi–Pb–Zn) which includes the Shizhuyuan W–Sn–Mo–Bi deposit (W: 80 Mt, Sn: 40 Mt, Bi: 20 Mt, Mo: 10 Mt)^21–23^. The zoned QGC was formed by successive Mesozoic magmatic intrusions^24–26^, which are assumed to have provided heat and metals for mineralization. However, the classification and geochronology of the QGC and the mineralization time of the Shizhuyuan deposit remain debated. Previous research on the QGC obtained intrusion ages ranging from 183 to 131 Ma^13,14,25–29^ and the mineralization ages from 145 to 160 Ma^28,30,31^. Thus, some phases of the QGC may indeed have a genetic relationship with the deposit, whereas other phases may not.

This study revisited the classification and dating of the QGC and mineralization to to clarify their genetic relationship. We present zircon LA-ICP-MS dating results for the QGC and combine these with petrology and field observations to constrain the structure and intrusive history of the QGC. Precise Ar–Ar dating of muscovite from the mineralized greisen vein allows for place constraints on the timing of the Shizhuyuan W–Sn–Mo–Bi deposit. Our results suggest close temporal relationship between the QGC and associated mineralization.

### Geological setting

**Figure 1:**
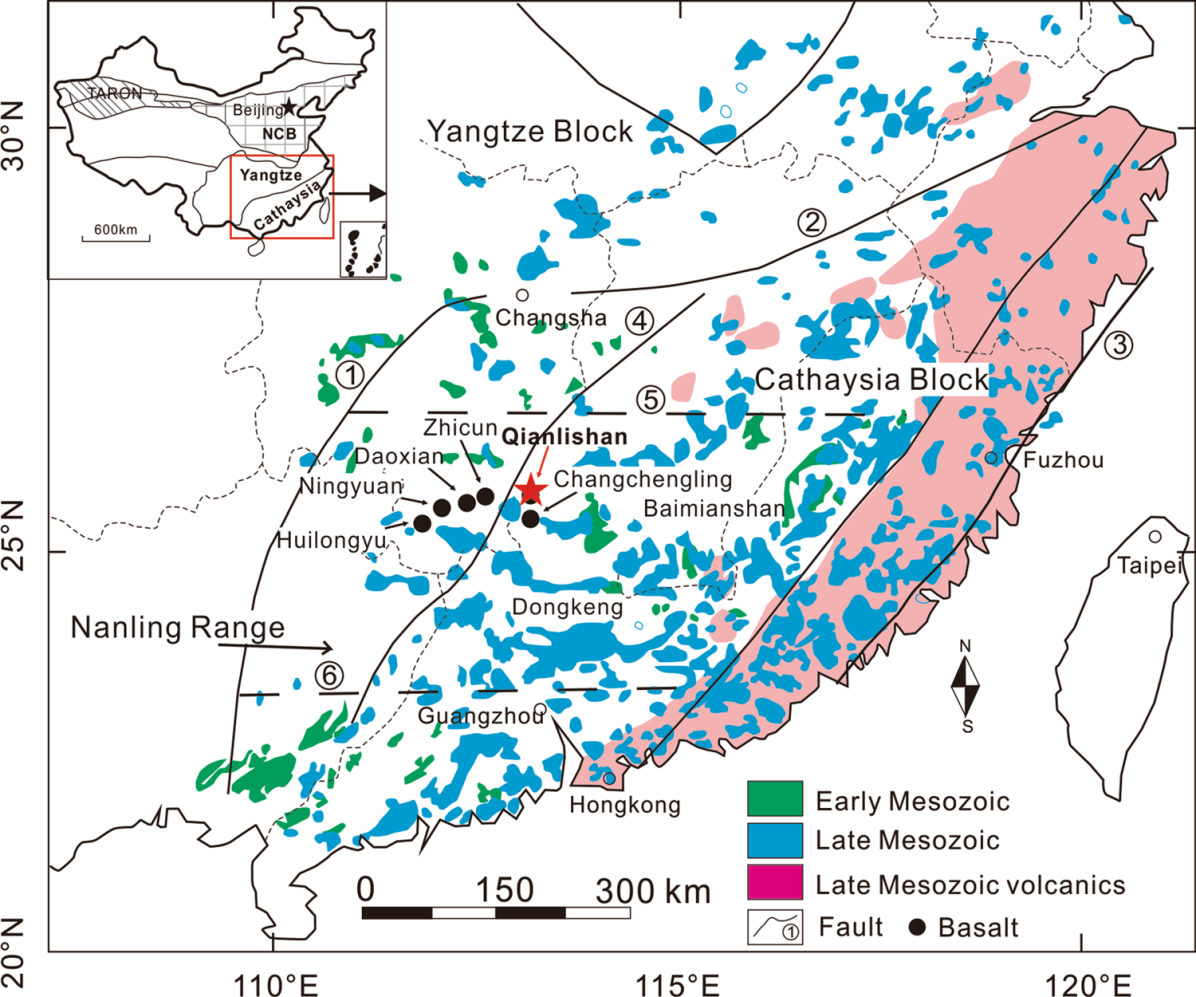
Geological map of the Nanling Range, South China (modified after Chen et al. (2016)^14^, copyright@ Elsevier, 2016). Faults: (**1**) Jiangshan–Shaoxing-Pingxiang Fault; (**2**) Zhenghe-Dapu Fault; (**3**) Changle-Nan’ao Fault; (**4**) Chenzhou-Linwu Fault; (**5**) Changlin-Guangchang buried Fault; (**6**) Wuzhou-Sihui buried Fault.

**Figure 2:**
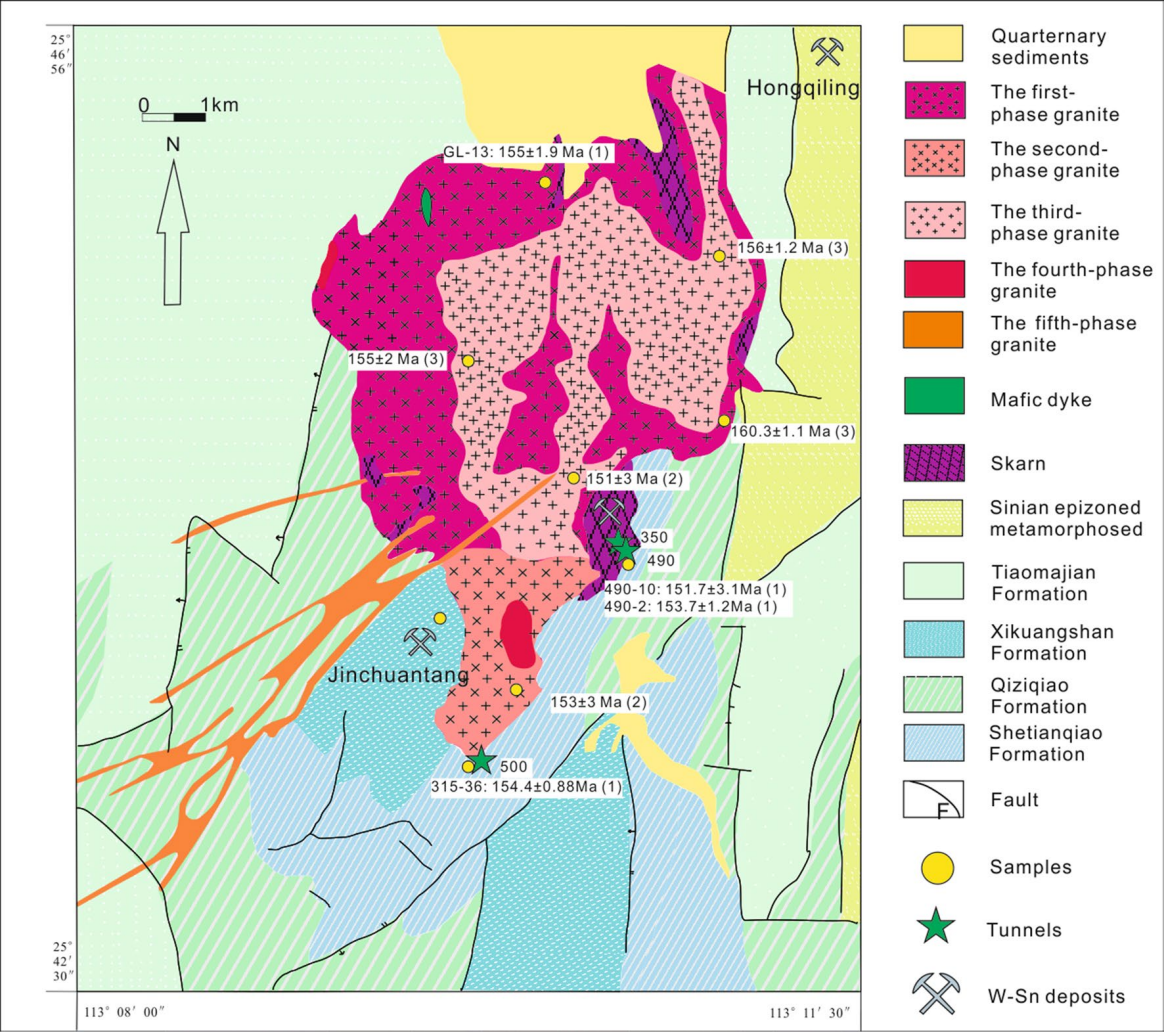
Schematic geological map of the Qianlishan district showing the QGC (modified after Chen et al. (2016)^14^, copyright@ Elsevier, 2016). The sample location and the obtained dates: (**1**) zircon LA-ICP-MS U–Pb dates from this study; (2) zircon SHRIMP U–Pb dates from Li et al. (2004)^28^; and (**3**) zircon LA-ICP-MS U–Pb dates from Chen et al. (2014)^13^.

The Nanling Range is located in the collision zone between the Yangtze Block and the Cathaysia Block (Fig. 1) where six main (buried) faults strike north and north-northeast. These blocks amalgamated during the early Neoproterozoic along the Qin-Hang deep fault zone^17,32,33^. This collision zone was reactivated in the early to late Mesozoic at 180–90 Ma, leading to the formation of numerous nonferrous and rare metal mineralized deposits, including W, Sn, Mo, Pb, Zn, U, Cu, Au, Ag and REEs^34^. The Shizhuyuan ore field is located approximately 16 km southeast of Chenzhou city, Hunan Province (Fig. 1). The faults are extensively developed in the ore field with strike directions of north-east, north–south and north-west (Fig. 2). The northeast and north–south faults control the distribution of the QGC and ambient mineral deposits. The Shizhuyuan ore field is located at the northern end of the Shizhuyuan-Taipingli synclinorium striking northeast. The ore field is surrounded by Sinian metasedimentary rocks, Devonian carbonate and clastic sedimentary rocks, the QGC and Quaternary sediments^35^ (Fig. 2). Sinian metasedimentary rocks occur only on the eastern edge of the ore field. These rocks are mostly weakly metamorphosed clastic sedimentary rocks. Specifically, the rocks comprise gray-green to gray-black, moderately thick, fine-grained quartz-rich sandstones, feldspathic sandstones, siltstones, phyllites and slate^36^. Devonian carbonate rocks and clastic sedimentary rocks are present as host rocks intensely altered by fracturing and magmatic activity (Fig. 2). From bottom to top, these rocks have been subdivided into four formations: (1) Tiaomajian Formation; (2) Qiziqiao Formation; (3) Shetianqiao Formation; and (4) Xikuangshan Formation. The first two formations belong to the Middle Devonian, and the last two to the Upper Devonian^30^. The Tiaomajian Formation (D_2_t) is > 358 m thick and occurs at the eastern and western sections of the ore field. It is mainly composed of gravel-bearing sandstones and conglomerates. The Qiziqiao Formation (D_2_q, > 520 m in thickness) occurs in the middle and southern parts of the ore field. It comprises micritic dolomites, limestones and dolomitic limestones. The Shetianqiao Formation (D_3_s, > 296 m in thickness), is present in the Shizhuyuan, Dongpo and Chaishan areas. It contains mainly banded micritic limestones. The Xikuangshan Formation (D_3_x, > 363 m in thickness) comprises thick-bedded limestones and dolomitic limestones containing flint concretions^37^. The Quaternary sediments are ~ 10 m thick, comprising slope wash. They are present only along the rivers in the northern part of the Shizhuyuan ore field.

### Petrography

**Figure 3:**
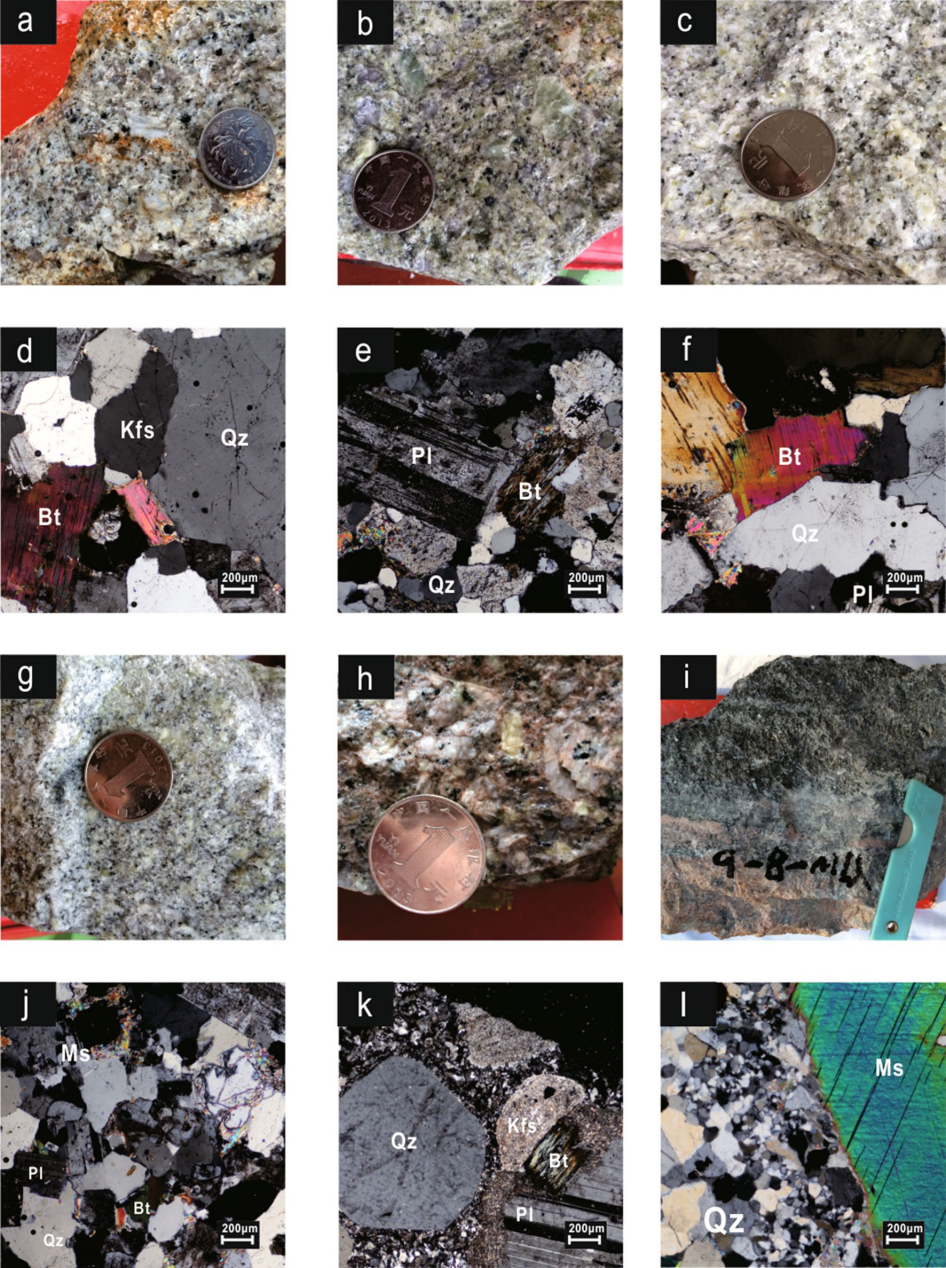
Photographs of the Qianlishan granites. (**a**, **d**) porphyritic biotite granite (Section 1: GL-13); (**b**, **e**) microfine-grained porphyritic biotite granite (Section 2: 315–36); (**c**, **f**) middle- to large-grained equigranular biotite granite (Section 3: 490–21); (**g**, **j**) fine-grained equigranular biotite granite (Section 4: 490–10); (**h**, **k**) granite porphyry dikes (Section 5: 490–2); (**i**, **l**): muscovite vein with mineralization. Abbreviations: Qz , Quartz; Pl,  Plagiarize; Bt, Biotite; Ms, Muscovite (Whitney DL and Evans BW et al. 2010)^53^.

Previous studies have indicated that the QGC (~ 10 km^2^) was intruded by lamprophyre and coeval mafic dikes. Since the mafic dikes are ~ 10 Ma younger than the QGC and the associated mineralization, they are not genetically related^38^. The QGC, which is spatially and temporally associated with W–Sn–Mo–Bi mineralization, can be subdivided into five Sections ^13,14,22,30,37–39^. *The first section (S*_*1*_*)* is a fine-grained porphyritic biotite granite (Fig. 3a,d) that outcrops within an area of ~ 4.0 km^2^ on the northern edge of the pluton. It is gray-white in color and comprises ~ 30 vol.% phenocrysts (2–7 mm in diameter), which are mostly potassium feldspar (~ 10 vol.%), plagioclase (~ 10 vol.%) and quartz (~ 10 vol.%), as well as minor biotite (~ 1 vol.%). The groundmass (0.3–1.0 mm in diameter) contains the same minerals. Dark inclusions within biotite, plagioclase, quartz and apatite occasionally occur in the S_1_ stocks and dikes. The accessory minerals in S_1_ are zircon, monazite, xenotime and ilmenite. *The second section (S*_*2*_*)* is a gray-white micro-fine-grained porphyritic biotite granite (Fig. 3b,e), occurring in the southern part of the pluton, with an outcrop area of ~ 1.1 km^2^. It contains phenocrysts of quartz (~ 17 vol.%) and feldspar (~ 13 vol.%) that range in size from 1 to 6 mm. The matrix is dominated by quartz, potassium feldspar, plagioclase and minor biotite (0.1–0.6 mm in diameter). Biotite is locally altered to chlorite (Fig. 3e). Accessory minerals in S_2_ are mainly zircon, monazite, xenotime, thorium, and apatite. *The third section (S*_*3*_*)* is a gray-white fine- to coarse-grained (mainly 0.3–0.8 mm in diameter) equigranular biotite granite (Fig. 3c,f) with an outcrop area of 4.4 km^2^. It contains quartz (~ 37 vol.%), plagioclase (~ 30 vol.%), alkali feldspar (~ 23 vol.%), biotite (~ 2 vol.%), and accessory minerals (< 3 vol.%) including zircon, monazite, and fluorite. Plagioclase (An = 0.01–0.03) occasionally exhibits overgrowth and argillization. *The fourth section (S*_*4*_*)* is a gray-white fine-grained (mostly 0.1–0.3 mm in diameter) equigranular two-mica granite (~ 0.1 km^2^; Fig. 3g,j), which comprises quartz (~ 40 vol.%), plagioclase (~ 31 vol.%), alkali feldspar (~ 22 vol.%), biotite (~ 2 vol.%), and muscovite (~ 1 vol.%). Plagioclase shows polysynthetic twinning, and alkali feldspar has Carlsbad twinning and perthitic texture. Primary and secondary muscovite were both found in this section. The primary muscovite grains present as euhedral intergranular sheets surrounded by plagioclase and quartz grains, whereas the secondary grains are distributed along the secondary fractures. The S_4_ has intruded into the first three sections, and pegmatite belts are often found on its top. The accessory minerals in this section are mainly zircon, thorite, topaz and fluorite. *The fifth section (S*_*5*_*)* represents a series of NE-striking (25–65°) granite porphyry dikes (Fig. 3h, 3k), comprising quartz (~ 15 vol.%), orthoclase (~ 8 vol.%), plagioclase (~ 15 vol.%) and minor biotite (~ 2 vol.%) phenocrysts (0.2–2 mm in diameter) within a matrix consisting mainly of quartz, orthoclase, plagioclase, and biotite. This section suffered strong alteration: (1) argillization was widely developed on the surface of plagioclase phenocrysts; (2) almost all biotite has been altered into chlorite and muscovite (Fig. 3k). Plagioclase comprises albite (An = 0.01 ~ 0.08) and andesine (An = 0.26 ~ 0.37). The Fe/(Fe + Mg) ratio of biotite is 0.74 ~ 0.75^22,40^. The accessory minerals mainly consist of euhedral to subhedral, prismatic allanite and apatite (60–150 μm × 10–60 μm wide), with a small number of zircon, monazite, fluorite, rutile and magnetite. All the studied zircon grains were generally wrapped in plagioclase, suggesting that they occurred as an early crystallization phase during crystal fractionation.

### Alteration and mineralization

#### Alteration

The alteration of the Shizhuyuan W–Sn–Mo–Bi deposit includes four types: a. skarnization; b. greisenization; c. marmarization; and d. feldspathization^22^.

##### Skarnization

The skarn located in the contact zone in the southeastern region of the QGC has experienced the most pervasive alteration in the Shizhuyuan deposit (Fig. 2). This skarn is approximately 1.2 km long, 1.0 km wide and 50–500 m thick (with an average thickness of 150–200 m). There are three types of skarns: a. original skarn; b. retrograde skarn; and c. veinlet skarn. The mineral assemblage of the skarn, whose parent rock is marble, comprises mainly garnet, pyroxene, idocrase and wollastonite^22^. The original skarn has been overprinted by a retrograde skarn. In comparison to the original skarn, the retrograde skarn contains much higher contents of fluorite, epidote, wolframite, scheelite, cassiterite, molybdenite, bismuthinite, magnetite, and pyrite^39^. Generally, mineralization occurs within the retrograde skarn rather than in the original skarn. Skarn veins crosscutting the margin of the retrograde skarn are tens to hundreds meters long and 10–50 cm wide. These skarn veins contain ores with grades of 1% to 6%^22^.

##### Greisenization

**Figure 4:**
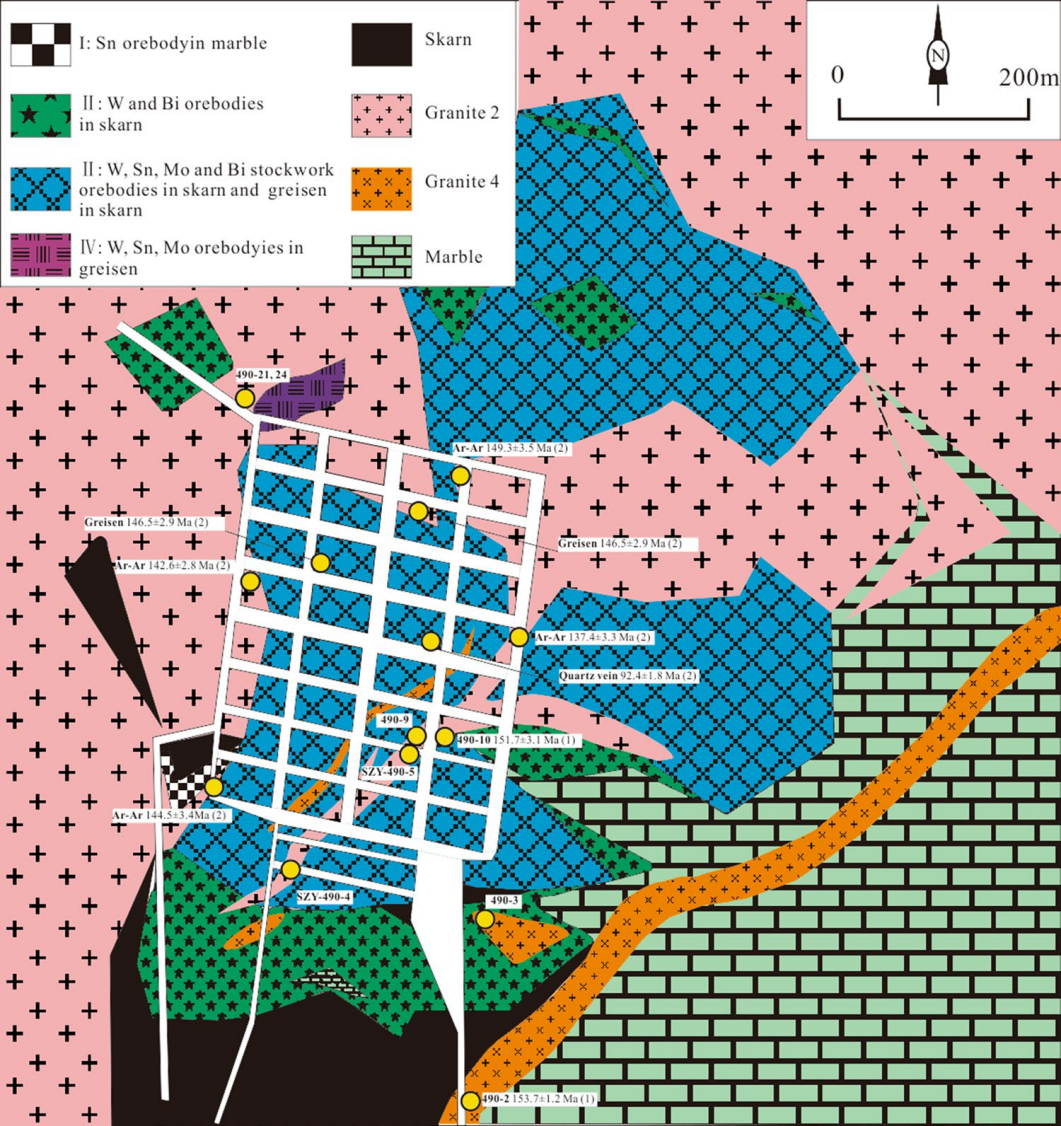
Sampling locations in Tunnel 490 (after Lu et al., 2003, copyright@Society of Economic Geologists, 2003)^39^. (**1**) zircon LA-ICP-MS U–Pb dates from this study; (**2**) muscovite Ar–Ar dates of the Qianlishan granites and Greisen from Yin et al., 2002^29^.

There are two types of greisen: massive greisen and vein-type greisen^21^. Massive greisen occurs mainly as discrete lenses in the upper section of the equigranular granites (S_3_ and S_4_); it contains quartz (~ 65%), mica (~ 16%), topaz (~ 8%), feldspar (~ 3%), chlorite (~ 2%) and fluorite (~ 1%). Compared with the massive greisen, the vein-type greisen has a similar mineral assemblage but with wider variations in mineral proportions: quartz (45–85%), mica (3–35%), topaz (5–40%), fluorite (2–10%), and feldspar (1–3%) as well as minor accessory minerals wolframite, scheelite, cassiterite, molybdenite, bismuthinite, magnetite, pyrite, and chalcopyrite (Fig. 3i,l). The vein-type greisen overlies the massive greisen, and it is distributed much more broadly (Fig. 4). Additionally, in Tunnel 490, a greisen vein is observed cutting through both the massive greisen and the skarn.

##### Marmarization

The stockwork marble vein, which is located at the contact between the overlying marble and the underlying skarn, is 750 m long, 300–600 m wide and 20–200 m thick. This vein comprises mainly fluorite, mica, tourmaline, and feldspar. The mineral grains are smaller than 0.05 mm in diameter^41^.

##### Feldspathization

Stockwork feldspar is a light-colored altered rock located in the fractures of the skarn. It contains mainly potassium feldspar and plagioclase and occasional quartz, fluorite, garnet, and pyroxene^42^.

#### Mineralization

Based on their compositions, textures and ore characteristics, the ores are clearly zoned. Pervasive greisenization plays the dominant role in defining these ore type classification. Mao et al. (1998) classified the ores into four types^26^. From top to bottom, these are *Type 1*—Sn–Cu ore within vein-type greisen superimposed on the porphyritic biotite granites (S_1_ and S_2_); *Type 2*—Sn–Be–Cu ore within the fine stockwork greisen overprinting the marble; *Type 3*—which W–Sn–Mo–Bi ore within thick stockwork greisen and rare stockwork greisen superimposed on the skarn; and *Type 4*—W–Sn–Mo–Bi ore within massive greisen at the top of the equigranular biotite granite stock (Fig. 4). Of these ores, the Type 3 ore has the greatest tonnage and represents the main mineralization stage^26,37^.

## Results

### Zircon LA-ICP-MS age and trace elements

**Figure 5:**
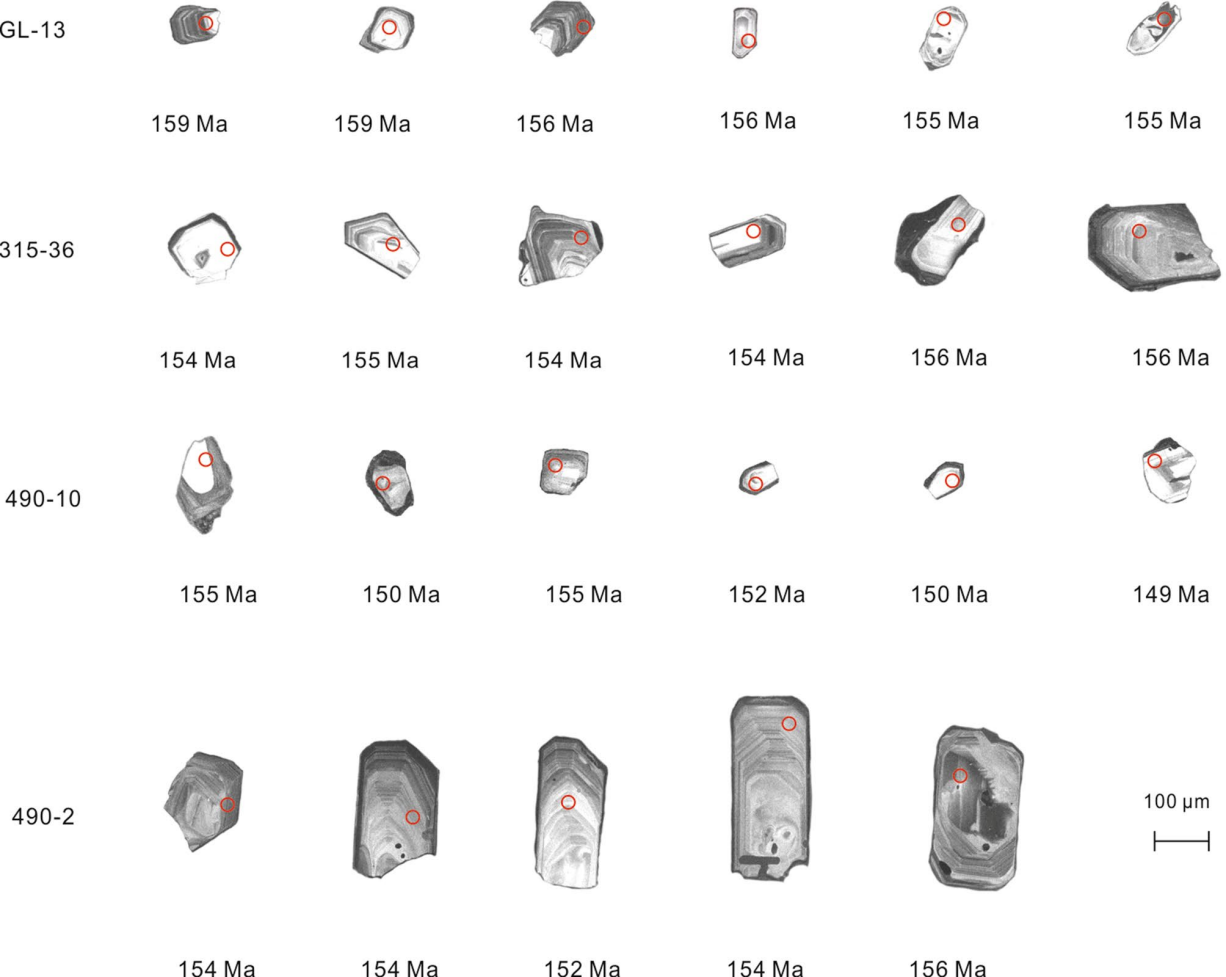
Cathodoluminescence images of zircons with corresponding ^206^Pb/^238^U ages for the samples from the QGC.

The zircons from Sample GL-13 (S_1_) are typically transparent, colorless to slightly brown, rectangular to prismatic crystals 100–150 μm long, with aspect ratios ranging from 2:1 to 3:1. Oscillatory zoning, with the occasional appearance of inherited cores, is common in these crystals (Fig. 5). The zircons from Sample 315–36 (S_2_) are mostly transparent, colorless to pale yellow, euhedral to subhedral crystals 100–200 μm long, with aspect ratios ranging from 2:1 to 3:1. The euhedral grains have concentric zoning with relatively bright cores in CL images (Fig. 5). Compared with those from the S_1_ and S_2_ granites, the zircons from the S_3_ granite (Sample 490–21) are similar in shape and color but are smaller (typically 50–100 μm long), with aspect ratios ranging from 2:1 to 1.5:1, and exhibit weak oscillatory zoning. The zircons of Sample 490–10 (S_4_) resemble those of Sample 490–21 in terms of their shape, color, and size (Fig. 5). The zircons of Sample 490–2 (S_5_) are characteristically long (100–250 μm), with aspect ratios ranging from 2:1 to 3:1. They are also transparent, colorless, and euhedral to subhedral. Oscillatory zoning is commonly visible in CL images (Fig. 5).

**Figure 6:**
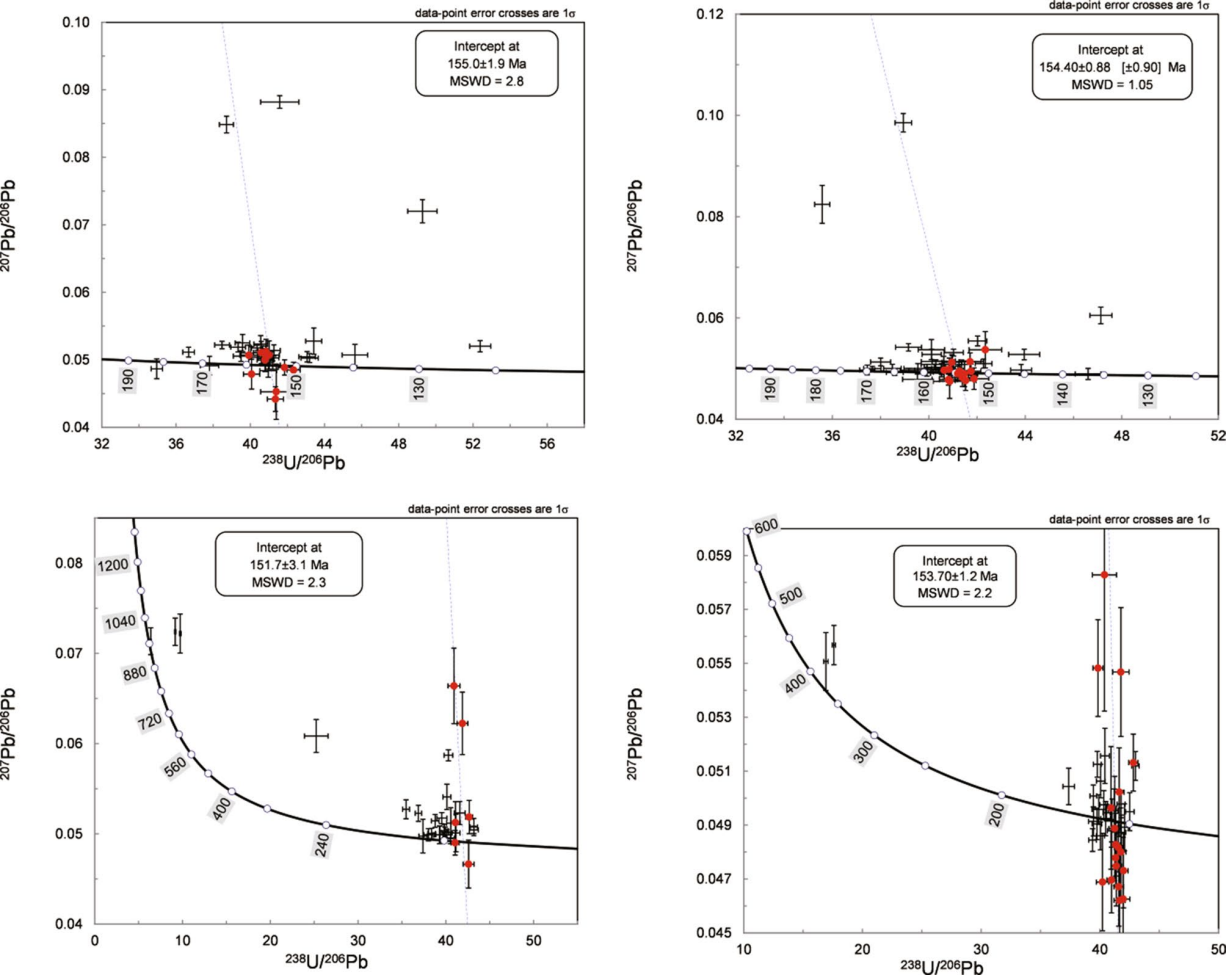
Zircon LA-ICP-MS U–Pb concordia diagrams for the QGC. The data from Sample 490 are too distributed to constrain either intercept age or concordant age. Data processing was carried out using concordia intercept ages on the Tera-Wasserburg plot utilizing ISOPLOT (Ludwig, v. 3.75, 2012, copyright@ BGC Berkeley Geochronology Center, 2006, available from: http://www.bgc.org/isoplot_etc/isoplot.html).

Zircon dates are summarized in Fig. 6 and Table 1. In total, 40 spots on zircon grains from Sample GL-13 were analyzed. Excluding the spots with abnormally high U content and associated with inherited zircons, 11 analyses yield a ^238^U/^206^Pb age of 155 ± 1.9 Ma (MSWD = 2.8, probability 0.002). Similarly, the weighted average ^238^U/^206^Pb age of Sample 315–36 (154.4 ± 0.88, MSWD = 1.05, probability 0.4) was obtained by pooling 14 analyses. The data for Sample 490–21 are too variable to constrain either an intercept age or a concordant age. Sample 490–10 has a slightly younger weighted average ^238^U/^206^Pb age (151.7 ± 3.1, MSWD = 2.3, probability 0.04) obtained by 6 analyses. Within error, this date is identical to those of Samples GL-13 and 315–36. Of forty analyses from Sample 490–2, 18 data points give a ^238^U/^206^Pb age of 153.7 ± 1.2 (MSWD = 2.2, probability 0.002). Trace element contents in zircon and calculated Ce anomalies and ‘Ti-in-zircon’ temperatures are presented in Table 2.

**Table 1:** LA-ICP-MS analyses of zircons in the QGC.

Spot	^204^Pb	Th	U	Th/U	^206^Pb/^238^U		^208^Pb/^232^Th		^207^Pb/^206^Pb		^206^Pb/^238^U	
	ppm	ppm	ppm	Ratio	Ratio	1 σ	Ratio	1 σ	Ratio	1 σ (%)	Age	1 σ
**Sample GL-13**	**155.0 ± 1.9 Ma**											
GL-13.1	< 0.017	718,990	10,659	67.5	0.0236	0.843	0.0071	49.9	0.0485	2.4	151	1.28
GL-13.2	< 0.011	730,641	6076	120.2	0.0239	0.829	0.0074	83.4	0.0489	2.3	152	1.27
GL-13.3	< 0.027	305,130	3704	82.4	0.0242	2.025	0.0084	202.6	0.0453	9.1	155	3.20
GL-13.4	< 0.022	1,026,513	13,159	78.0	0.0244	1.318	0.0075	91.1	0.0507	4.1	155	2.07
GL-13.5	< 0.014	190,508	2582	73.8	0.0242	1.048	0.0080	82.2	0.0442	4.2	155	1.65
GL-13.6	< 0.012	401,861	3321	121.0	0.0245	1.059	0.0076	95.6	0.0512	3.5	156	1.67
GL-13.7	< 0.015	561,004	9162	61.2	0.0245	1.030	0.0083	56.2	0.0502	3.7	156	1.63
GL-13.8	< 0.016	393,170	4171	94.3	0.0245	1.048	0.0074	95.4	0.0500	3.4	156	1.66
GL-13.9	< 0.016	257,419	3246	79.3	0.0246	1.035	0.0076	72.1	0.0511	3.2	157	1.64
GL-13.10	< 0.019	949,154	11,431	83.0	0.0250	1.101	0.0073	75.6	0.0479	4.6	159	1.79
GL-13.11	< 0.013	372,898	6020	61.9	0.0251	1.191	0.0080	60.1	0.0507	3.0	159	1.90
**Sample 315–36**	**154.4 ± 0.88 Ma**											
315–36.1	< 0.013	201,314	2221	90.6	0.0245	1.671	0.0072	139.8	0.0475	7.1	156	2.67
315–36.2	< 0.011	257,224	2669	96.4	0.0240	1.085	0.0075	85.3	0.0514	3.4	152	1.67
315–36.3	< 0.015	189,337	2953	64.1	0.0242	1.030	0.0075	68.7	0.0482	3.6	154	1.61
315–36.4	< 0.013	208,855	2992	69.8	0.0241	1.044	0.0074	67.7	0.0476	4.1	154	1.64
315–36.5	0.0160	296,177	3674	80.6	0.0241	1.319	0.0079	117.9	0.0489	5.2	154	2.07
315–36.6	< 0.012	163,950	3379	48.5	0.0243	0.929	0.0077	49.4	0.0490	3.0	155	1.45
315–36.7	< 0.012	306,397	3946	77.7	0.0244	1.008	0.0074	60.5	0.0512	3.0	155	1.58
315–36.8	< 0.013	788,172	3769	209.1	0.0240	0.984	0.0075	134.5	0.0495	2.9	153	1.51
315–36.9	< 0.011	311,522	5131	60.7	0.0242	0.960	0.0075	50.6	0.0494	2.6	154	1.49
315–36.10	< 0.019	278,724	6415	43.4	0.0236	1.590	0.0077	93.3	0.0537	6.7	150	2.46
315–36.11	< 0.015	462,909	6012	77.0	0.0245	1.130	0.0076	94.4	0.0499	4.5	156	1.80
315–36.12	< 0.011	362,534	6403	56.6	0.0245	0.917	0.0077	39.7	0.0478	2.2	156	1.44
315–36.13	< 0.017	414,235	6364	65.1	0.0246	0.847	0.0076	57.8	0.0497	3.5	157	1.36
315–36.14	< 0.018	526,273	9600	54.8	0.0239	1.455	0.0078	87.6	0.0480	4.4	152	2.24
**Sample 490–10**	**151.7 ± 3.1 Ma**											
490–10.1	0.022	206	280	0.7	0.0244	1.670	0.0088	102.5	0.0664	6.3	152	2.65
490–10.2	< 0.015	321	356	0.9	0.0239	1.455	0.0085	103.0	0.0622	5.6	150	2.26
490–10.3	0.012	404	469	0.9	0.0234	0.949	0.0074	63.1	0.0519	3.6	149	1.44
490–10.4	< 0.014	264	581	0.5	0.0243	1.267	0.0084	50.2	0.0513	4.5	155	1.99
490–10.5	< 0.011	238	767	0.3	0.0244	1.101	0.0079	25.3	0.0490	2.8	155	1.71
490–10.6	< 0.026	1181	1019	1.2	0.0235	1.453	0.0074	114.6	0.0466	5.7	150	2.22
**Sample 490–2**	**153.7 ± 1.2 Ma**											
490–2.1	< 0.012	156,345	2488	62.8	0.0240	1.136	0.0076	72.7	0.0462	3.8	153	1.76
490–2.2	< 0.014	132,686	2967	44.7	0.0239	1.649	0.0078	80.2	0.0547	4.4	151	2.52
490–2.3	< 0.010	184,902	2909	63.6	0.0239	1.042	0.0072	65.2	0.0480	3.4	153	1.61
490–2.4	< 0.011	145,320	2979	48.8	0.0240	0.974	0.0079	54.9	0.0502	3.3	153	1.51
490–2.5	< 0.014	269,537	2962	91.0	0.0242	1.189	0.0076	100.9	0.0489	4.0	154	1.86
490–2.6	< 0.014	183,598	3902	47.0	0.0238	1.342	0.0076	80.8	0.0463	5.0	152	2.07
490–2.7	< 0.013	199,402	3431	58.1	0.0251	1.037	0.0085	50.5	0.0548	3.3	159	1.67
490–2.8	< 0.011	201,265	3677	54.7	0.0242	1.058	0.0075	51.8	0.0475	3.1	154	1.64
490–2.9	< 0.012	205,122	3943	52.0	0.0238	0.924	0.0075	54.9	0.0473	2.9	152	1.42
490–2.10	< 0.011	221,015	4274	51.7	0.0240	0.961	0.0077	46.5	0.0467	3.1	154	1.49
490–2.11	< 0.011	259,883	4825	53.9	0.0244	0.922	0.0077	44.3	0.0470	2.6	156	1.45
490–2.12	< 0.026	379,376	6085	62.3	0.0248	2.517	0.0099	123.2	0.0583	8.7	156	4.02
490–2.13	< 0.011	281,035	5602	50.2	0.0242	0.907	0.0075	44.8	0.0483	2.4	154	1.41
490–2.14	< 0.018	251,636	5120	49.1	0.0244	1.073	0.0081	69.1	0.0496	4.6	155	1.71
490–2.15	< 0.017	370,518	7611	48.7	0.0249	1.301	0.0077	64.3	0.0469	3.9	159	2.08
490–2.16	< 0.011	315,444	6794	46.4	0.0242	0.854	0.0075	38.8	0.0478	2.2	154	1.32
490–2.17	< 0.011	834,605	7440	112.2	0.0234	0.898	0.0072	76.3	0.0513	2.1	148	1.34
490–2.18	< 0.010	608,213	9788	62.1	0.0241	0.833	0.0077	41.0	0.0482	1.9	153	1.28

**Table 2:** Zricon Trace element compositions, Ce anomalies, and Ti-in-zircon temperatures.

Spot	Ti	La	Ce	Pr	Nd	Sm	Eu	Gd	Tb	Dy	
**Sample GL-13**											
GL-13.1	4.7	< 0.0005	35	0.2	2.6	6.5	0.6	29.2	10.5	135	
GL-13.2	7.2	0.02	37.5	0.3	4.9	10	1.5	51.3	16.1	186.2	
GL-13.3	9.3	0.01	27	0.1	2	3.4	0.4	18	6.9	80.3	
GL-13.4	10.9	0.05	32.9	0.3	5.8	8.2	1.2	40.8	13.3	146.7	
GL-13.5	12.3	0.05	18.8	0.1	1.3	3	0.5	16.6	5.4	67.8	
GL-13.6	15.5	0.02	30.1	0.3	3.7	7.9	1.5	39.8	12.4	141.6	
GL-13.7	5.4	2.45	36.8	1	5	2.8	0.3	20.5	7.1	90.5	
GL-13.8	13.1	0.03	28.2	0.2	4.8	9.5	1.6	41.8	13.7	165.1	
GL-13.9	11.6	< 0.001	24.7	0.1	1.5	3.7	0.9	22	7.1	85.9	
GL-13.10	6.1	0.03	31.9	0.2	2.8	7.3	0.7	41.9	13.1	165.3	
GL-13.11	8.7	0.01	24	0.1	1.1	2.8	0.3	16.1	5.9	77.7	
**Sample 315–36**											
315–36.1	9	0.01	24.2	0	0.9	2.6	0.4	14.6	5.1	65.6	
315–36.2	10.7	0.01	27.9	0.1	1.8	4.6	0.7	21.9	7.2	86.9	
315–36.3	11.6	< 0.0004	22.1	0.1	1.1	2.5	0.4	13.7	5	58.7	
315–36.4	13.8	0.01	16.5	0.1	2	4.8	0.8	23.3	7.6	90.7	
315–36.5	11.5	0.05	18.7	0.1	2.3	5.1	0.8	25.1	7.7	92.4	
315–36.6	10.6	0.01	14.9	0	0.7	1.8	0.4	12.3	4.2	53.6	
315–36.7	8.1	< 0.0004	25.4	0.1	1.4	3	0.4	19.3	6.3	79.8	
315–36.8	11.6	0.11	47.1	0.5	7.9	13.3	2.7	67.1	19.3	218.5	
315–36.9	5.7	0	22.4	0.1	1.5	2.9	0.2	17.4	5.8	70.1	
315–36.10	6	0.03	19.3	0.1	0.5	1.6	0.1	11.9	4.6	59.3	
315–36.11	7.2	< 0.001	30.1	0.1	2	3.9	0.2	23.1	7.6	91.6	
315–36.12	6.9	0.02	30.5	0	1	2.7	0.1	13.3	5.1	64	
315–36.13	7.6	0.27	22.4	0.1	1.5	3.5	0.2	20.6	7.5	93.3	
315–36.14	6.8	0.13	25.3	0.1	1.5	3.2	0.2	19.9	7.1	94.9	
**Sample 490–10**											
490 − 10.1	10.7	0.06	26.5	0.1	2.7	4.8	0.9	26.2	9	100.6	
490–10.2	11.1	0.09	27.1	0.4	5.2	9.3	1.8	45	14.1	170.4	
490–10.3	13.1	0.1	37.3	0.2	3.3	5.7	0.9	32.3	10.5	123.4	
490–10.4	7.8	0.04	27.4	0.1	0.9	1.6	0.2	14.5	4.9	67.9	
490–10.5	9.3	0.03	21.8	0.2	2.4	4	0.8	20.6	7.3	96.4	
490–10.6	16.8	0.19	70.7	1	14.3	23	3.1	102.4	31.7	367.3	
**Sample 490–2**											
490–2.1	12.4	0.01	6	0.1	2.4	5.8	0.6	27.3	9.7	114.4	
490–2.2	8.1	0.04	8.3	0	0.4	1.2	0.1	7.5	3	38.9	
490–2.3	10	0	13.8	0	0.8	2.3	0.3	14.4	4.5	63.3	
490–2.4	12.2	0	8	0	0.7	1.5	0.3	9	3.3	41.2	
490–2.5	14.1	0.03	14.9	0.1	2.3	5.6	0.7	26.8	8.6	104.9	
490–2.6	10.3	< 0.0009	7.6	0	0.5	1.6	0.2	12.2	4.3	47.9	
490–2.7	10.8	0.08	9.5	0.1	1.2	2.9	0.3	15.9	5.6	69.8	
490–2.8	11.3	0.01	10.1	0	0.8	2.3	0.3	12.4	5	59.9	
490–2.9	13.1	0	10.4	0	1	2.2	0.3	11.6	4.4	54.2	
490–2.10	8.6	< 0.0007	11.6	0	0.9	2.5	0.2	13	4.8	61.5	
490–2.11	10.6	0	13	0	0.9	2.6	0.3	13.8	5.2	59.3	
490–2.12	8.3	0.36	15.4	0.5	3.3	5.3	0.4	23.8	8.4	99.4	
490–2.13	8.2	0	12.9	0	0.7	2	0.3	14.4	5	65.5	
490–2.14	7.7	0.46	15.9	0.2	1.3	1.6	0.1	12.2	4.2	58.2	
490–2.15	9.6	0	14.1	0.1	0.7	2.5	0.3	11.9	4.4	58.2	
490–2.16	8	0	14.2	0	0.9	2.2	0.1	10.8	3.9	49.2	
490–2.17	21	0.08	12.9	0.6	9.3	18.4	2.4	97.3	28.3	306.9	
490–2.18	6.4	0.01	16.4	0.1	1.8	4.4	0.2	22.6	8.1	104.5	

Spot	Ho	Er	Tm	Yb	Lu	δCe	T(°C)	lg(fO_2_)	δFMQ	T(K)	10^4^/T (1/K)
**Sample GL-13**											
GL-13.1	50.4	248.9	54.1	506.2	95.6	49.4	679	− 17.0	0.3	951.8	10.5
GL-13.2	64	292.7	58.2	509.9	95	20.2	716	− 18.3	− 1.9	989.1	10.1
GL-13.3	30.5	134.3	28.4	266.4	47.1	47.2	739	− 14.0	1.9	1011.9	9.9
GL-13.4	55.8	276	60.5	562.2	109.8	19.7	754	− 16.5	− 1.0	1026.9	9.7
GL-13.5	24.6	114.6	23.6	218	41.6	47	766	− 12.6	2.6	1039.0	9.6
GL-13.6	49.8	231.8	45.6	410.1	78.6	21.9	789	− 14.4	0.3	1062.0	9.4
GL-13.7	34.5	165.9	36.5	332.2	62.5	37.5	690	− 17.4	− 0.3	963.5	10.4
GL-13.8	58.2	270.6	55.8	475.8	91.8	16.4	772	− 16.3	− 1.2	1044.7	9.6
GL-13.9	31.4	145.6	30.9	273.4	52.8	54.1	760	− 12.4	3.0	1032.9	9.7
GL-13.10	60	281.9	57.5	509.9	95.4	35.7	701	− 17.0	− 0.2	974.1	10.3
GL-13.11	28.7	142.2	30.2	270.2	50.8	87.6	733	− 11.9	4.1	1005.9	9.9
**Sample 315–36**											
315–36.1	24.4	115.9	24.9	230.6	44.5	130.9	736	− 10.3	5.7	1009.3	9.9
315–36.2	33.2	156.4	33.1	300.8	59.5	67.8	753	− 11.9	3.6	1025.6	9.8
315–36.3	22	103.4	22.1	202.2	37.5	96	760	− 10.2	5.1	1033.4	9.7
315–36.4	31.4	148.1	29.8	264.5	49.6	32	777	− 13.6	1.4	1049.8	9.5
315–36.5	32.6	150.2	30.4	273.5	49.5	30.8	760	− 14.5	0.8	1032.5	9.7
315–36.6	19.9	101.3	20.6	196.2	37.2	114.5	751	− 10.0	5.6	1024.3	9.8
315–36.7	29.3	143.4	30.2	276.7	52.3	92.5	727	− 12.1	4.1	999.6	10.0
315–36.8	74.3	331.3	65.5	567.7	106	19.6	760	− 16.2	− 0.8	1032.9	9.7
315–36.9	26.3	125.5	26.5	242.9	45.7	76	696	− 14.4	2.5	969.3	10.3
315–36.10	22	113.8	24.9	240.3	46.1	270.5	701	− 9.4	7.4	973.7	10.3
315–36.11	33.6	161.1	32.1	304.4	58.1	71.6	715	− 13.6	2.8	988.4	10.1
315–36.12	23.9	113	23.8	220.3	41.7	147.5	712	− 11.1	5.4	985.2	10.2
315–36.13	36.6	170.7	36.7	332.6	65.3	82.6	721	− 12.8	3.5	993.9	10.1
315–36.14	35.6	172.9	37.9	356.2	67.7	103.4	710	− 12.5	4.0	983.3	10.2
**Sample 490–10**											
490 − 10.1	37.6	177.7	39	343.8	64.6	71.5	752	− 11.7	3.8	1025.4	9.8
490–10.2	59.7	272.6	57.1	491.4	95.2	32.4	756	− 14.5	0.9	1029.2	9.7
490–10.3	45.4	209.8	43.4	377.4	72.3	78.3	772	− 10.4	4.7	1044.9	9.6
490–10.4	26.8	136.1	29.9	285.8	54.7	367.1	723	− 7.1	9.2	995.5	10.0
490–10.5	36.5	186.9	40.4	383.2	73.5	84.6	739	− 11.8	4.1	1011.9	9.9
490–10.6	122.8	555.5	102.5	871.4	157.7	24.4	797	− 13.6	0.9	1070.1	9.3
**Sample 490–2**											
490–2.1	41.9	191.1	38.6	343.2	62.3	13.9	766	− 17.2	− 2.0	1039.4	9.6
490–2.2	16.2	76.6	16.7	155.8	28.6	167.3	727	− 9.8	6.3	1000.0	10.0
490–2.3	22.7	108.2	21.7	200.2	36.8	116.5	746	− 10.2	5.5	1019.0	9.8
490–2.4	15	73.4	15.7	141.2	27.1	90.4	765	− 10.2	5.0	1038.2	9.6
490–2.5	35.5	157.9	30.8	275.9	49.1	31.4	779	− 13.5	1.4	1052.3	9.5
490–2.6	19.1	92.5	20.1	182.6	34.7	121	749	− 9.9	5.7	1021.6	9.8
490–2.7	25.1	119.2	24.5	221.7	40.4	49.8	753	− 13.1	2.5	1025.9	9.7
490–2.8	22.5	107.6	23.1	205.7	38.9	88.6	757	− 10.7	4.7	1030.3	9.7
490–2.9	19.4	96.1	20.1	180.5	33.9	75.4	772	− 10.6	4.5	1044.7	9.6
490–2.10	22.5	109.4	23.3	208.5	39.3	88.1	732	− 12.0	4.1	1004.9	10.0
490–2.11	22.2	108.7	22.5	201.8	38.2	98.7	751	− 10.6	5.0	1024.4	9.8
490–2.12	34.1	156.1	32.9	292.2	49.8	25.6	728	− 16.8	− 0.7	1001.4	10.0
490–2.13	23.8	119.1	25.6	233.6	45.1	149.8	728	− 10.2	5.9	1001.0	10.0
490–2.14	21.1	104.5	21.9	206.7	39.8	116.6	722	− 11.5	4.8	994.7	10.1
490–2.15	22.5	108.3	23	217.6	39.6	141.4	742	− 9.7	6.1	1015.3	9.8
490–2.16	18.5	94	20.6	192.9	35.7	123.4	725	− 11.1	5.1	998.3	10.0
490–2.17	100.3	425.4	79.6	644.5	114.9	5.2	821	− 18.4	− 4.3	1093.9	9.1
490–2.18	39.1	187.9	39.3	361	66	64.6	706	− 14.5	2.2	979.0	10.2

Notes: Ce anomalies (δCe) are calculated based on Nd, Sm, and Gd to Lu using the lattice strain model (Blundy and Wood, 1994)^55^.

### Whole-rock major and trace element chemistry

**Figure 7:**
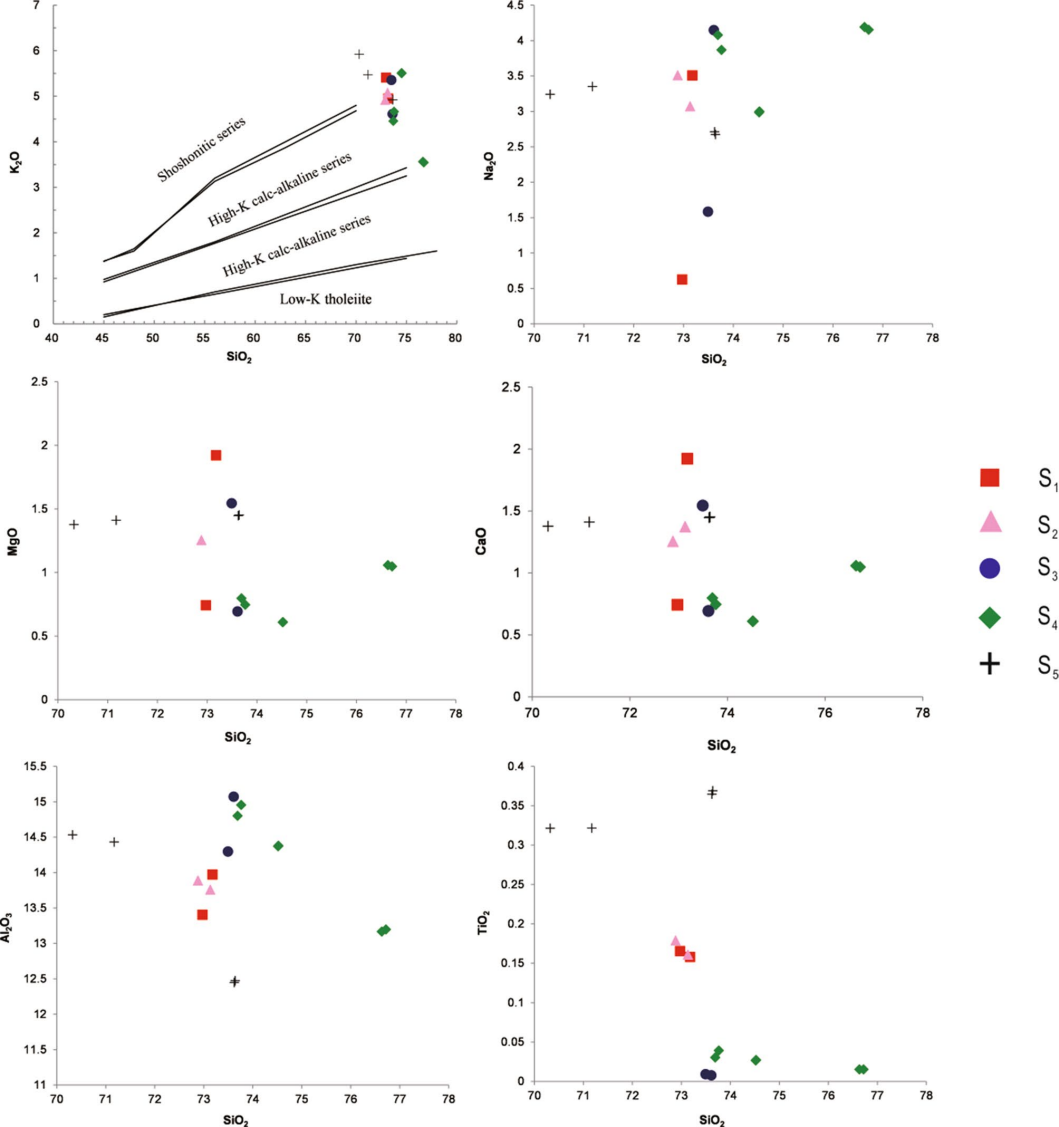
Harker diagram for the QGC.

**Figure 8:**
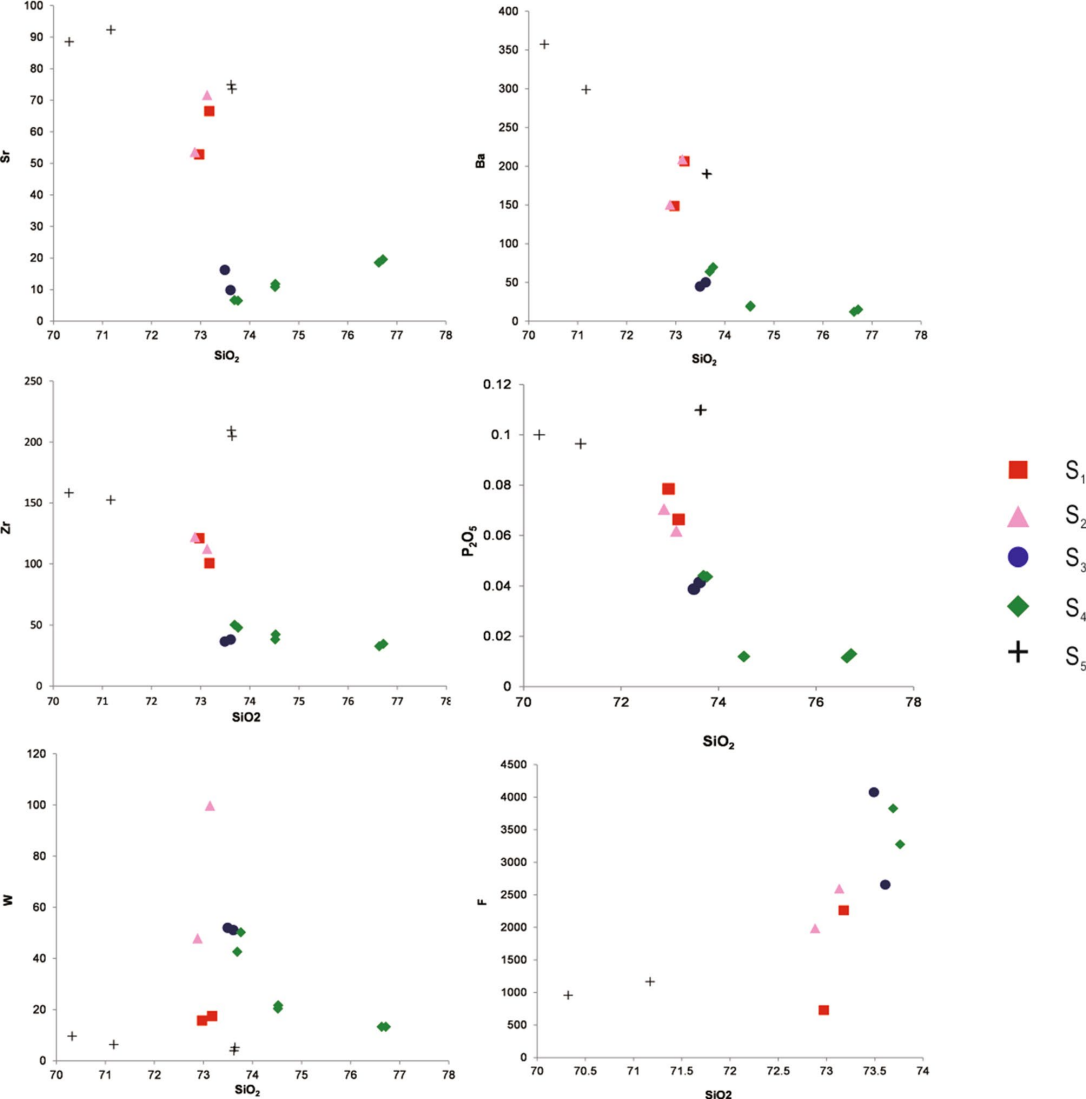
SiO_2_ vs Sr, Ba, Zr, P_2_O_5_, W and F for the QGC.

**Figure 9:**
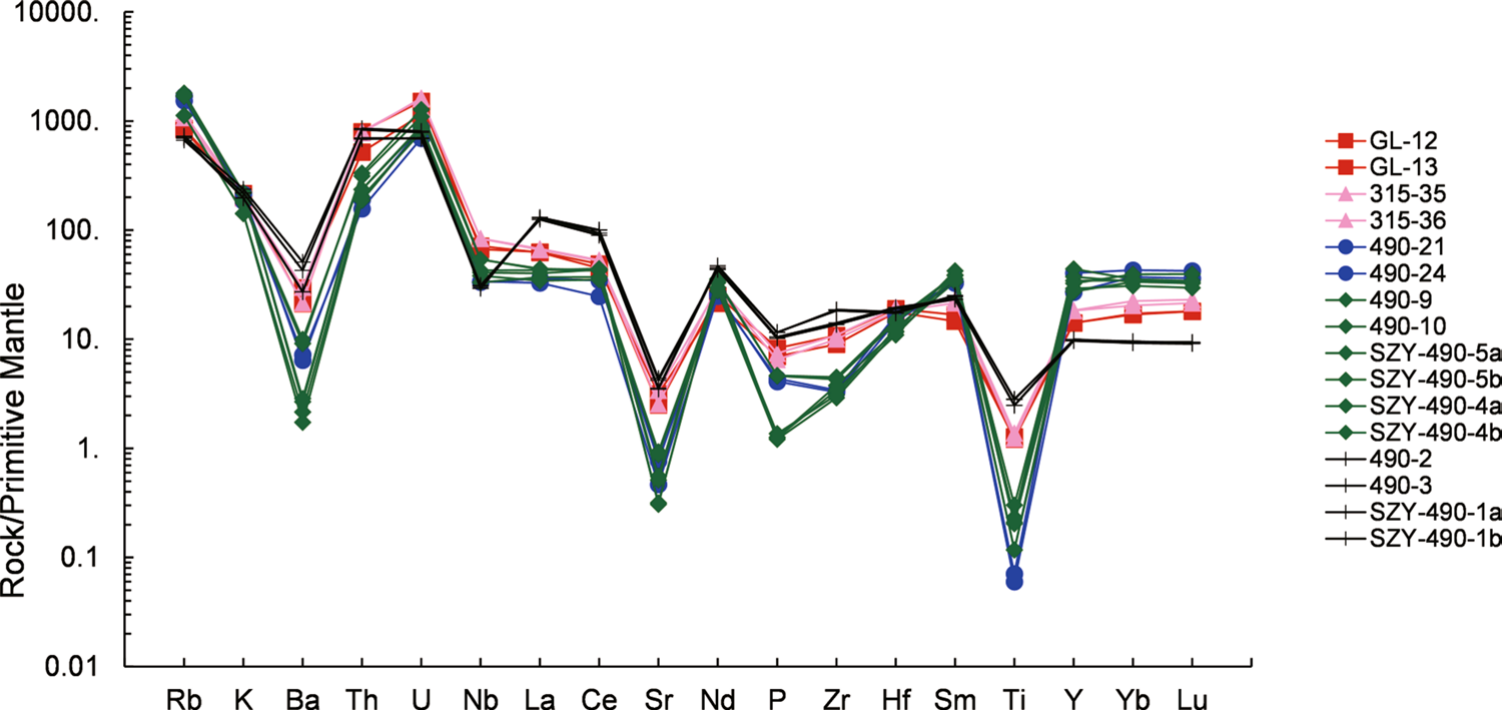
REE patterns for the QGC.

**Figure 10:**
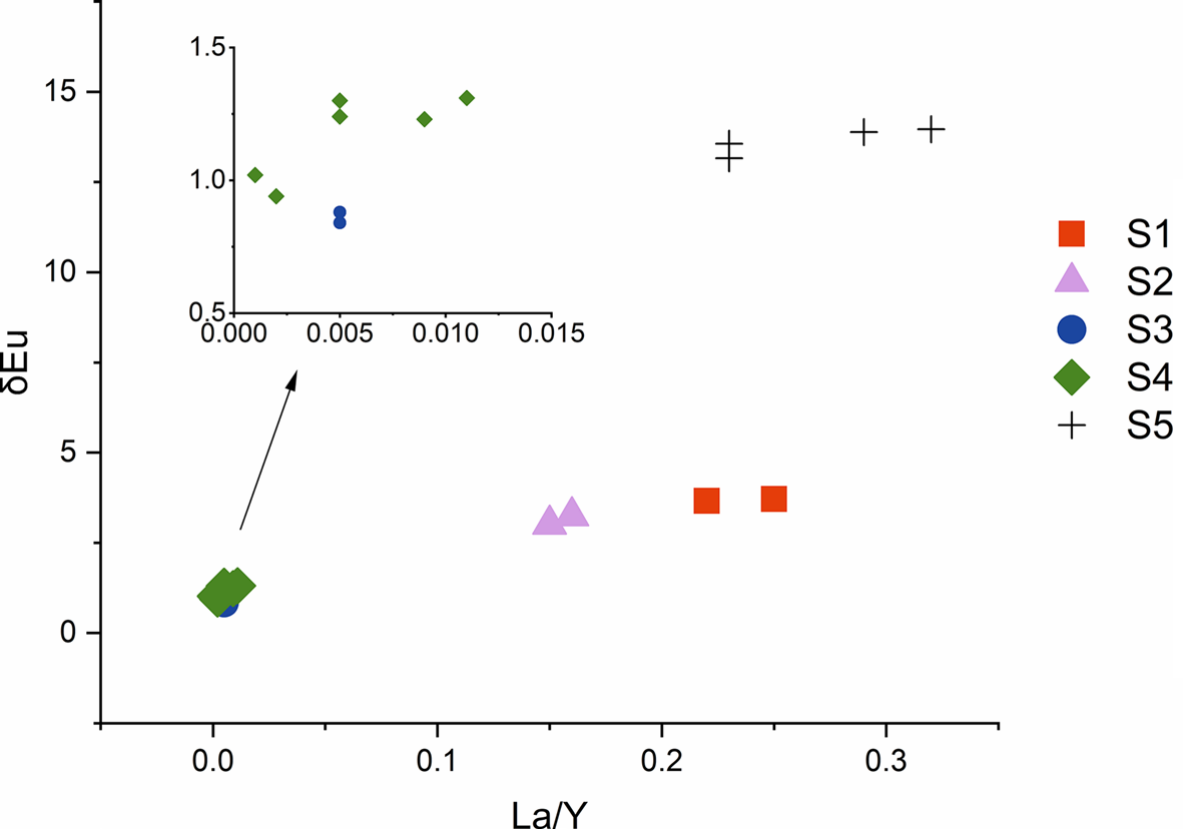
La/Y vs δEu for the QGC.

Twenty representative samples were analyzed for their major and trace element compositions (Table 3). These samples are characterized by high SiO_2_ (70.32–78.28 wt%) and K_2_O (3.54–5.92 wt%) contents. Most of the samples plot in the fields of high-K calc-alkaline field, whereas seven samples plot in the shoshone field. The aluminum saturation index (ASI) values of the five phases of Qianlishan granites are 0.95–1.78, 1.04–1.05, 0.88–1.34, 1.04–1.2, and 1–1.2. The S_1_, S_2_, and S_5_ porphyritic granites have higher contents of K_2_O, CaO, MgO, TiO_2_, Zr, Sr, Ba, and P_2_O_5_ but lower contents of Na_2_O and Al_2_O_3_ than the other granites (Figs. 7, 8). Notably, fluorine concentrations of the S_3_ and S_4_ equigranular biotite granites (2500–10,400 ppm) are much higher than those of the S_5_ granite porphyry (< 2000 ppm), whereas the S_1_ and S_2_ porphyritic biotite granites have intermediate F contents (2000–6800 ppm). Moreover, S_2_, S_3_ and S_4_ have relatively high W contents (20–100 ppm), whereas S_1_ and S_5_ have lower W contents (< 20 ppm). In the primitive mantle-normalized diagrams (Fig. 9), S_3_ and S_4_ exhibit the strongest negative Ba, Sr, P, and Ti anomalies, whereas S_1_ and S_2_ show much smaller anomalies. S_5_ has trace element patterns similar to those of S_1_ and S_2_. As presented in Fig. 10 and Table 3, S_3_ and S_4_ have the lowest La/Yb (0.08–1.31) and δEu (0.002–0.011), followed by S_1_ and S_2_ with La/Yb (3.01–3.71) and δEu (0.15–0.25). S_5_ has the highest La/Yb (13.16–13.96) and δEu ((0.23–0.32).

**Table 3:** Major and trace element composition of samples from the main intrusive stages of the QGC.

Phase	LLD (%)	Phase 1								
Section		Section 1		Section 2		Section 3				
Sample		GL-12	GL-13	315–35	315–36	490–21	490–24			
SiO_2_ (%)	19	72.97	73.18	73.13	72.88	73.61	73.49			
Al_2_O_3_	0.3	13.41	13.97	13.76	13.89	15.07	14.30			
TFe_2_O_3_	0.31	3.83	1.17	1.38	1.42	0.825	1.07			
MgO	0.2	1.89	0.255	0.279	0.319	0.090	0.268			
CaO	0.1	0.743	1.92	1.37	1.26	0.694	1.54			
Na_2_O	0.3	0.627	3.51	3.07	3.51	4.15	1.58			
K_2_O	0.1	5.41	4.95	5.08	4.92	4.61	5.36			
MnO	0.02	0.074	0.025	0.047	0.027	0.044	0.088			
TiO_2_	0.02	0.166	0.158	0.161	0.179	0.008	0.009			
P_2_O_5_	0.01	0.079	0.066	0.062	0.070	0.041	0.039			
LOI	0.0001	0.610	0.833	1.71	1.61	0.917	2.33			
FeO	0.18	1.03	0.65	0.795	1.05	0.67	0.862			
Li (ppm)	7.8	67.1	50.1	67.7	63.5	192	29.7			
Be	0.27	12.0	11.5	15.6	7.32	7.75	19.5			
Sc	1.8	8.05	7.52	8.39	8.62	5.76	6.56			
V	11.5	11.2	11.0	10.5	10.9	1.77	2.04			
s	2	2.56	2.58	1.97	4.24	1.44	1.20			
Co	2.4	3.33	0.76	1.06	0.99	0.091	0.13			
Ni	4.9	2.85	1.61	1.25	2.13	1.26	0.97			
Cu	6.4	7.12	3.14	3.67	4.46	1.31	1.65			
Zn	16	19.1	14.5	15.1	18.0	17.5	24.0			
Ga	2.1	19.7	19.5	20.2	20.6	33.8	31.1			
Ge	0.1									
Rb	9.6	541	532	744	683	1072	976			
Sr	48	52.8	66.6	71.6	53.5	9.85	16.2			
Zr	21	121	101	112	122	38.2	36.6			
Nb	1.9	47.5	51.0	59.5	59.7	24.1	23.8			
Mo	0.22	12.0	1.26	2.22	1.77	1.52	0.52			
Cd	0.027									
In	0.024	0.039	0.034	0.097	0.11	0.086	0.097			
Sb	0.88	0.16	0.094	0.094	0.077	0.077	0.11			
Te	0.02									
Cs	0.72	26.8	24.4	27.9	24.8	33.9	23.8			
Ba	31	149	207	209	150	50.2	45.0			
Hf	0.54	5.89	5.49	5.57	6.06	5.14	4.47			
Ta	0.124	8.02	7.84	10.7	10.8	22.8	16.5			
W	0.24	15.8	17.6	99.7	47.9	51.1	52.0			
Tl	0.09	2.28	2.16	3.87	3.77	4.65	4.96			
Pb	7.6	27.9	24.7	38.5	41.0	38.5	26.6			
Bi	0.055	10.2	1.93	0.84	0.59	7.22	5.82			
Th	1.5	67.8	44.1	66.3	68.2	13.3	16.1			
U	0.67	31.9	24.0	34.2	33.4	14.6	18.1			
Y	3.3	64.3	64.1	83.1	83.8	123	184			
La	4.8	43.3	42.9	45.4	46.3	22.6	24.7			
Ce	8.7	87.1	79.6	92.8	94.1	43.9	61.3			
Pr	1.2	9.97	8.66	11.1	11.6	8.46	8.84			
Nd	3.9	34.2	28.9	39.6	42.0	33.7	35.7			
Sm	0.8	7.43	6.48	9.54	10.1	14.5	14.7			
Eu	0.17	0.53	0.54	0.50	0.48	0.022	0.023			
Gd	0.67	7.33	6.66	9.27	9.75	13.6	15.4			
Tb	0.11	1.49	1.40	1.96	2.07	3.61	4.12			
Dy	0.54	9.57	9.08	12.6	13.2	23.2	27.2			
Ho	0.1	2.04	1.98	2.66	2.77	4.43	5.34			
Er	0.34	6.60	6.50	8.25	8.87	13.5	19.7			
Tm	0.053	1.16	1.13	1.41	1.54	2.48	2.93			
Yb	0.3	8.48	8.31	10.1	11.0	18.4	21.2			
Lu	0.046	1.34	1.33	1.59	1.70	2.68	3.14			
La/Y		3.66	3.71	3.24	3.01	0.88	0.84			
δEu		0.22	0.25	0.16	0.15	0.005	0.005			

Phase	Phase 2						Phase 3			
Section	Section 4						Section 5			
Sample	490–9	490–10	SZY-490-5a	SZY-490-5b	SZY-490-4a	SZY-490-4b	490–2	490–3	SZY-490-1a	SZY-490-1b
SiO_2_ (%)	73.76	73.69	76.63	76.72	74.52	74.52	70.32	71.17	73.64	73.62
Al_2_O_3_	14.96	14.80	13.17	13.20	14.38	14.37	14.53	14.43	12.47	12.45
TFe_2_O_3_	0.836	0.953	0.046	0.030	0.40	0.39	2.33	2.37	0.88	0.86
MgO	0.104	0.107	0.11	0.11	0.055	0.067	0.466	0.456	0.43	0.43
CaO	0.746	0.797	1.06	1.05	0.61	0.61	1.38	1.41	1.45	1.45
Na_2_O	3.87	4.08	4.19	4.15	3.00	2.98	3.24	3.35	2.67	2.71
K_2_O	4.66	4.45	3.56	3.54	5.50	5.51	5.92	5.47	4.92	4.92
MnO	0.034	0.036	0.007	0.008	0.022	0.022	0.039	0.040	0.041	0.041
TiO_2_	0.039	0.030	0.015	0.015	0.027	0.026	0.321	0.322	0.37	0.36
P_2_O_5_	0.044	0.044	0.012	0.013	0.012	0.012	0.100	0.096	0.110	0.110
LOI	1.00	1.06	1.10	1.05	1.04	1.06	1.48	1.01	1.47	1.48
FeO	0.556	0.63	0.088	0.10	0.41	0.42	1.705	1.724	1.48	1.49
Li (ppm)	123	132	8.89	10.5	13.3	11.8	18.6	20.6	35.0	35.4
Be	67.7	12.1	6.27	6.43	22.7	21.4	5.98	6.48	6.61	6.76
Sc	9.20	8.97	4.16	4.66	5.42	5.43	6.68	6.79	5.01	5.35
V	3.10	2.60	1.31	2.20	0.82	2.53	21.3	21.8	18.9	20.7
s	1.71	1.79	2.36	2.68	3.05	2.38	4.16	4.27	4.56	5.47
Co	0.12	0.11	0.11	0.10	0.17	0.18	3.32	3.14	3.59	3.57
Ni	1.00	0.81	0.26	0.43	0.62	0.25	2.50	2.45	1.79	2.29
Cu	1.65	3.69	11.4	11.1	1.69	1.54	5.56	5.11	4.93	5.13
Zn	14.8	12.7	0.81	0.81	16.0	15.5	44.5	41.9	48.9	53.1
Ga	32.1	30.2	24.9	29.6	29.5	27.5	20.6	20.2	19.4	20.2
Ge			2.67	2.69	2.52	2.40			1.38	1.31
Rb	1071	1072	712	713	1144	1088	463	424	441	453
Sr	6.48	6.68	18.5	19.6	11.8	10.8	88.5	92.3	73.5	74.9
Zr	47.9	50.2	32.6	34.6	42.3	38.2	158	153	205	210
Nb	38.3	38.6	27.2	23.6	30.5	29.1	21.0	20.8	22.2	21.8
Mo	4.93	3.33	9.67	10.0	2.01	2.13	3.62	8.28	2.20	2.72
Cd			0.083	0.080	0.076	0.038			0.044	0.069
In	0.18	0.14	0.022	0.023	0.14	0.13	0.052	0.044	0.038	0.037
Sb	0.30	0.20	0.11	0.11	0.16	0.14	0.094	0.094	0.12	0.11
Te			0.025	0.019	0.025	0.011			0.025	0.025
Cs	49.3	23.0	8.67	8.52	28.8	27.5	13.8	13.2	13.5	13.5
Ba	69.6	63.8	12.1	15.1	20.0	18.7	357	299	190	191
Hf	4.44	4.67	3.60	3.88	3.66	3.39	6.05	5.88	5.35	5.51
Ta	14.9	14.9	23.8	29.2	13.3	15.6	2.71	2.69	2.64	2.82
W	50.2	42.6	13.3	13.3	21.7	20.4	9.67	6.36	5.27	3.91
Tl	4.38	4.59	2.35	2.53	3.76	3.72	2.48	2.10	1.82	1.84
Pb	54.3	52.1	19.4	20.4	39.2	39.3	35.7	37.8	30.9	31.8
Bi	5.30	10.7	1.77	1.01	0.73	0.80	0.36	0.42	0.36	0.33
Th	26.6	28.3	15.9	17.1	20.0	20.1	70.8	72.7	58.1	59.4
U	22.9	26.8	17.5	16.7	18.3	18.9	16.5	16.9	14.7	14.5
Y	170	201	125	148	157	133	44.0	45.3	44.1	44.9
La	30.5	30.0	23.6	25.4	29.6	27.6	88.7	90.0	85.9	89.4
Ce	76.3	75.6	62.5	66.0	78.6	77.0	177	179	159	167
Pr	11.6	10.9	9.48	10.3	11.8	11.3	18.3	18.6	17.9	18.6
Nd	46.7	44.7	37.6	40.3	48.3	45.4	59.5	58.8	61.4	64.0
Sm	17.1	17.2	14.9	16.0	18.8	17.1	10.3	10.3	10.9	11.2
Eu	0.06	0.051	0.007	0.009	0.019	0.016	1.02	0.93	0.78	0.77
Gd	16.3	17.7	13.0	7.02	7.90	7.01	9.53	9.49	9.35	9.61
Tb	3.99	4.33	3.60	3.85	4.16	3.78	1.48	1.48	1.58	1.60
Dy	25.3	27.9	22.2	24.4	25.2	23.4	7.97	8.10	8.39	8.42
Ho	4.90	5.48	4.31	4.67	4.85	4.27	1.55	1.59	1.57	1.62
Er	14.3	15.8	12.5	14.1	14.0	12.5	4.57	4.80	4.60	4.76
Tm	2.41	2.56	2.62	3.07	2.74	2.48	0.69	0.71	0.82	0.82
Yb	16.6	17.5	16.6	19.4	17.1	15.2	4.56	4.65	4.68	4.73
Lu	2.41	2.53	2.44	2.94	2.43	2.19	0.67	0.70	0.69	0.69
La/Y	1.31	1.23	1.02	0.94	1.24	1.30	13.96	13.88	13.16	13.56
δEu	0.011	0.009	0.001	0.002	0.005	0.005	0.32	0.29	0.23	0.23

LOI, Loss on ignition; LLD, Lower limit of detection.

### Muscovite Ar–Ar age

**Figure 11:**
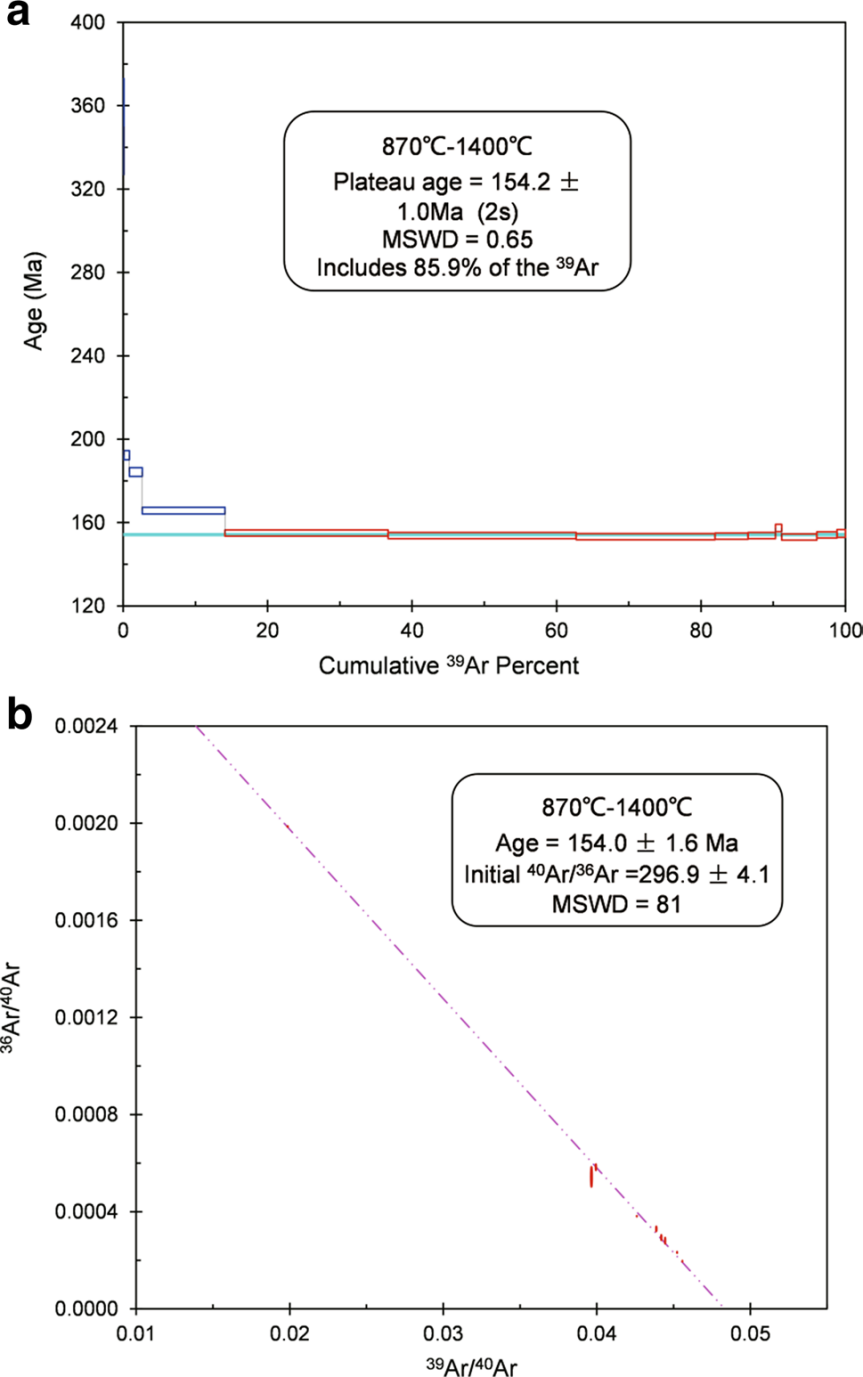
Plateau (**a**) and isochron (**b**) Ar–Ar age of muscovite from mineralizing greisen in the Shizhuyuan deposit. ISOPLOT software (Ludwig, v. 3.75, 2012, copyright@ BGC Berkeley Geochronology Center, 2006, available from: http://www.bgc.org/isoplot_etc/isoplot.html) was used for data processing.

The Ar–Ar analytical data of Sample YJW-8-B are summarized in Table 4. Age spectra and inverse isochrons are plotted in Fig. 11.

**Table 4:** Ar–Ar stepwise heating data for muscovite samples from the QGC and the Shizhuyuan deposit.

T	(^40^Ar/^39^Ar)_m_	(^36^Ar/^39^Ar)_m_	(^37^Ar_0_/^39^Ar)_m_	(^38^Ar/^39^Ar)_m_	^40^Ar(%)	40Ar*/39Ar	^39^Ar	Age	± 1σ
(°C)							(Cum.) (%)	(Ma)	(Ma)
Sample YJW-8-B									
700	2073.448	6.8485	0.0000	1.3186	2.4	49.7199	0.1	350	23
760	58.7071	0.1103	0.0000	0.0376	44.48	26.1126	0.87	192.3	2.2
800	35.7398	0.0364	0.0000	0.0215	69.87	24.9724	2.63	184.3	2.1
840	27.9391	0.0189	0.0000	0.0175	79.96	22.3392	14.1	165.7	1.6
870	23.4786	0.009	0.0000	0.0156	88.7	20.8253	36.67	155	1.5
900	21.9468	0.0043	0.0000	0.0148	94.16	20.6644	62.73	153.8	1.5
930	22.1158	0.0051	0.0000	0.015	93.11	20.5911	81.93	153.3	1.5
960	22.4984	0.0063	0.0000	0.0157	91.67	20.6253	86.51	153.5	1.5
1000	22.6302	0.0066	0.0000	0.0154	91.3	20.662	90.32	153.8	1.5
1040	25.2233	0.0137	0.0000	0.0149	83.91	21.1641	91.16	157.4	1.7
1080	22.7963	0.0075	0.0000	0.0158	90.22	20.5658	96.05	153.1	1.5
1200	25.0391	0.0146	0.0000	0.017	82.71	20.7107	98.83	154.1	1.5
1400	50.3407	0.0999	0.0000	0.0332	41.32	20.8003	100	154.8	1.9

Muscovite from Sample YJW-8-B yields a plateau age of 154.2 ± 1.0 Ma (MSWD = 0.65) (Fig. 11a). All errors are quoted at the 2σ level. The plateau age comprises nine steps accounting for 85.9% of the total 39Ar released and agrees with the inverse isochron age of 154.0 ± 1.6 Ma (MSWD = 0.81) (Fig. 11b). The estimated initial ^40^Ar/^36^Ar is 296.9 ± 4.1%, which is identical to the present-day initial ^40^Ar/^36^Ar (295.5%). The characteristics of the spectra suggest the absence of argon loss and excess argon. In other words, the Ar–Ar system of the muscovite remained closed during the geological history of Sample YJW-8-B.

## Discussion

### Reclassification of granitic phases

Zoned plutons often require numerous magmatic intrusion pulses continuously emplaced over millions of years, because individual magmatic pulses commonly last for less than 100,000 years^7,9,10,24,43^. The QGC exhibits normal zoning with the most differentiated phases (S_3_ and S_4_) in the central part. Each phase can be distinguished by their emplacement age, mineral assemblage and geochemistry. Different classification schemes for the QGC have been suggested. According to some studies, the equigranular biotite granite (S_3_) and the porphyritic biotite granite (S_1_) have been classified as the QGC^25,28,37,39^. In contrast, other studies combined the porphyritic biotite granite (S_1_) with the porphyritic biotite granite (S_2_) that is located on the southern margin of the QGC^13,14,22,31,38^. Additionally, the porphyritic biotite granite (S_1_) has been considered a separate phase by Guo et al. (2015)^27^. To clarify the relationship between different rock types with the QGC, we have undertaken a systematic study of petrology, geochronology and geochemistry of all five sections of the pluton.

**Figure 12:**
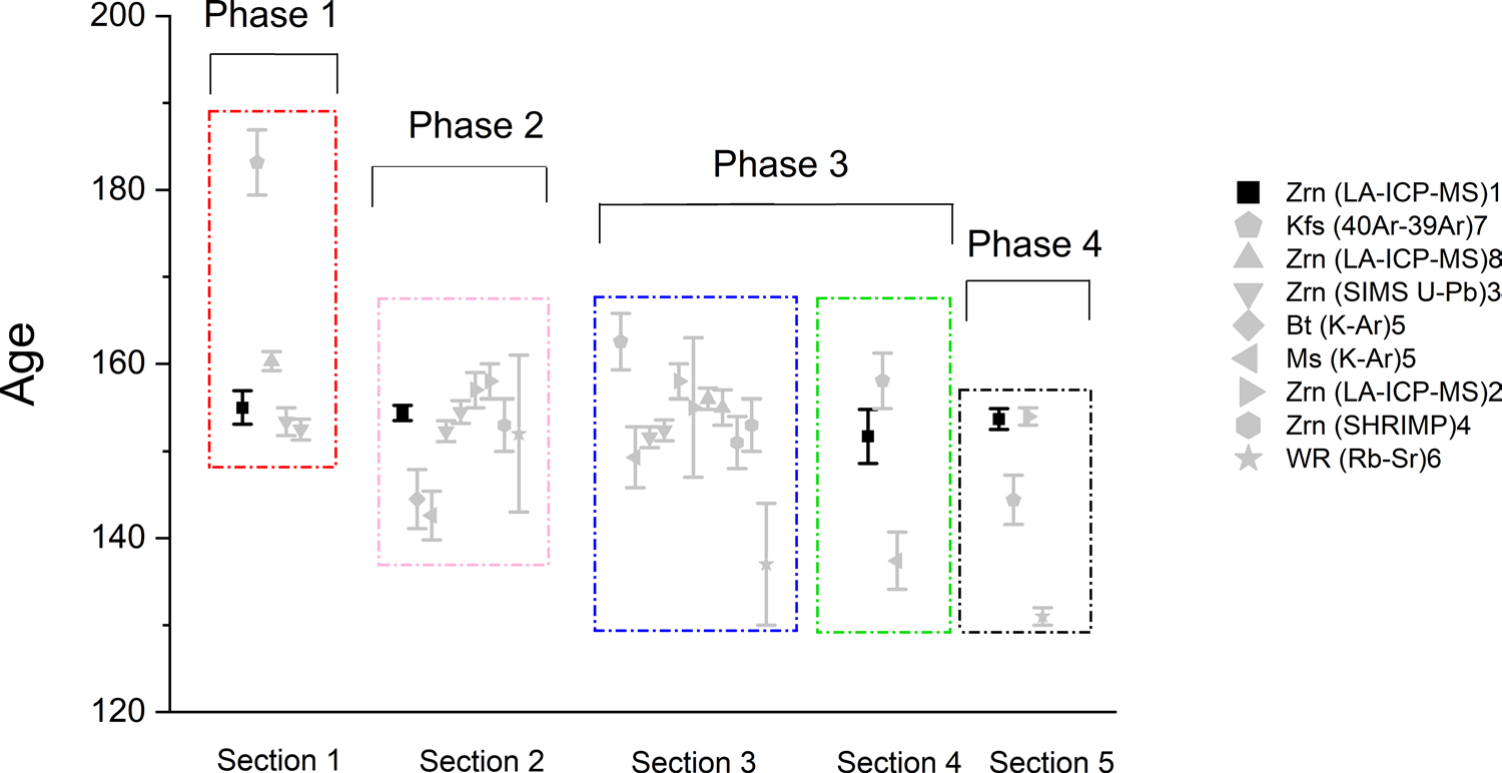
Summary of the dating of the QGC from previous publications and this study. Zrn , Zirco; Kfs, K-feldspar; Bt, Biotite; Ms, Muscovite (Whitney DL and Evans BW et al. 2010)^53^. WR , whole-rock. Reference: 1. This study; 2. Chen et al. 2016^14^; 3. Guo et al. 2015^27^; 4. Li et al. 2004^28^; 5. Yin et al. 2002^29^; 6. Mao et al. 1998^26^; 7. Liu et al. 1997^25^; 8. Chen et al. 2014^13^.

Previous geochronological investigations have used diverse methods and obtained a variety of results (Table 5, Fig. 12). For the *porphyritic biotite granite (P*_*1*_*),* Liu et al. (1997) obtained a potassium feldspar ^40^Ar/^39^Ar plateau age of 183.17 ± 3.75 Ma^25^. In contrast, Chen et al. (2016) obtained two zircon U–Pb ages of 157 ± 2 Ma and 158 ± 2 Ma using laser ablation inductively coupled plasma mass spectrometry (LA-ICP-MS)^14^. Similarly, Chen et al. (2014) obtained two zircon U–Pb ages of 160 ± 1 Ma and 156 ± 1 Ma using LA-ICP-MS^13^. Furthermore, Guo et al. (2015) obtained four younger ages based on zircon analyses using secondary ion mass spectrometry (SIMS): 153.4 ± 1.6 Ma, 152.5 ± 1.2 Ma, 154.5 ± 1.3 Ma and 152.3 ± 1.2 Ma, which were identical (within error) to the zircon ages of 153 ± 3 Ma^27,28^ determined using sensitive high-resolution ion microprobe (SHRIMP). Overall, the previous ages of the P_1_ granite range from 183.2 to 152.3 Ma.

**Table 5:** Ages of the QGC from this study and the literature.

Phase	Section	Lithology	Mineral for dating	Method	Age (Ma)	Age
1	1	Fine-grained porphyritic biotite grantie	Zircon	(LA-ICP-MS)^1^	155 ± 1.9	155
			K feldspar	(^40^Ar-^39^Ar)^7^	183.17 ± 3.75	183.17
			Zircon	(LA-ICP-MS)^8^	160.3 ± 1.1	160.3
			Zircon	(SIMS U–Pb)^3^	153.4 ± 1.6; 152.5 ± 1.2	153.4
	2	Microfine-grained porphyritic biotite grantie	Zircon	(LA-ICP-MS)^1^	154.4 ± 0.88	154.4
			Biotite	(K–Ar)^5^	144.5 ± 3.4	144.5
			Muscovite	(K–Ar)^5^	142.6 ± 2.8	142.6
			Zircon	(SIMS U–Pb)^3^	154.5 ± 1.3;152.3 ± 1.2	154.5
			Zircon	(LA-ICP-MS)^2^	157 ± 2; 158 ± 2	157
			Zircon	SHRIMP^4^	153 ± 3	153
			whole-rock	(Rb–Sr)^6^	152 ± 9	152
2	3	Medium- and coarse- grained equigranular biotite grantie	K feldspar	(^40^Ar-^39^Ar)^7^	162.55 ± 3.25	162.55
			Muscovite	(K–Ar)^5^	149.3 ± 3.5	149.3
			Zircon	(SIMS U–Pb)^3^	152.4 ± 1.2; 151.6 ± 1.2	152.4
			Zircon	(LA-ICP-MS)^2^	158 ± 2; 155 ± 8	158
			Zircon	(LA-ICP-MS)^8^	156 ± 1.2; 155 ± 2	156
			Zircon	SHRIMP^4^	151 ± 3	151
			whole-rock	(Rb–Sr)^6^	137 ± 7	137
	4	Fine-graied equigranular biotite grantie	Zircon	(LA-ICP-MS)^1^	151.7 ± 3.1	151.7
			K feldspar	(^40^Ar-^39^Ar)^7^	158.07 ± 3.16(Pegmatite)	158.07
			Muscovite	(K–Ar)^5^	137.4 ± 3.3	137.4
3	5	Granite porphyry dykes	Zircon	(LA-ICP-MS)^1^	153.7 ± 1.2	153.7
			K feldspar	(^40^Ar-^39^Ar)^7^	144.41 ± 2.83	144.41
			Zircon	(LA-ICP-MS)^2^	154 ± 1	154
			whole-rock	(Rb–Sr)^6^	131 ± 1	131

Reference: 1. This study; 2. Chen et al. (2016)^14^; 3. Guo et al.(2015)^27^; 4. Li et al. (2004)^28^; 5. Yin et al. (2002)^29^; 6. Mao et al. (1998)^26^; 7. Liu et al. (1997)^25^; 8. Chen et al. (2014)^13^.

The *equigranular biotite granite (P*_*2*_*)* can be classified into two groups. (1) One group consists of medium- and coarse-grained equigranular biotite granite, for which Liu et al. (1997) obtained a potassium feldspar ^40^Ar/^39^Ar plateau date of 162.55 ± 3.25 Ma^25^. In addition, Chen et al. (2016) obtained two slightly younger zircon U–Pb ages of 157 ± 2 Ma and 158 ± 2 Ma using LA-ICP-MS, which agreed with the younger zircon LA-ICP-MS U–Pb age of 155 ± 2 Ma obtained by Chen et al. (2014)^13,14^. However, Li et al. (2004) obtained a SHRIMP zircon U–Pb age of 151 ± 3 Ma, which was consistent with the muscovite ^40^Ar/^39^Ar age of 149.3 ± 3.5 Ma obtained by Yin et al. (2002)^28,29^. (2) The other group is fine-grained equigranular biotite granite, for which Yin et al. (2002) obtained a muscovite ^40^Ar/^39^Ar age of 137.4 ± 3.3 Ma^29^. In addition, Chen et al. (2016) did not classify the equigranular granite into two groups with distinct grain sizes, and they obtained two zircon U–Pb ages of 158 ± 2 Ma and 155 ± 1 Ma using LA-ICP-MS^14^. In summary, the P_2_ granite was emplaced at 137.4–162.6 Ma.

For the *granite porphyry (P*_*3*_*),* Chen et al. (2016) obtained a zircon U–Pb age of 154 ± 1 Ma^14^. Liu et al. (1997) obtained a potassium feldspar ^40^Ar/^39^Ar plateau date of 144.41 ± 2.83 Ma^25^. Together, these ages indicate that the emplacement of the P_3_ granite occurred at 154.0–144.4 Ma.

Bulk-rock Rb–Sr ages and potassium/mica (K/Ar)-Ar isochron ages are not reliable for dating the emplacement of granitic plutons because they can be affected by thermal disturbances caused either by prolonged fluid convection or tectonic processes^14,44,45^. In contrast, zircon U–Pb dating, which has closure temperatures of 700–800°C^46,47^, is a reliable tool for placing geochronological constraints on plutonic emplacement.

Here, LA-ICP-MS was used to carry out zircon U–Pb dating. Our new data indicate that the S_1_, S_2,_ S_4_, and S_5_ granites were emplaced at 155 ± 1.9 Ma, 154.4 ± 0.88 Ma, 151.7 ± 3.7 Ma and 153.7 ± 1.2 Ma, which are identical within error (Table 5, Fig. 12).

The QGC (Si content > 70%) was formed by a felsic magma and experienced advanced differentiation, with a fractionation index of > 92^22^. During the fractionation process of felsic magma, although major elements have a limited range, trace elements that favor feldspars (Rb, Sr, Ba) and accessory minerals (Th, P, U, Zr, Sn, Ce, Y) show wide variation^48,49^. The variation diagrams (Figs. 7, 8) indicate that the contents of Na_2_O and Al_2_O_3_ increase from the S_1_ to S_4_ granites, whereas Ca and Mg decrease. The decrease in P with SiO_2_ content indicates fractionation of apatite. This is consistent with the presence of apatite in Sample GL-2 (S_1_) and the absence of apatite in Sample 490–10 (S_4_). The depletions in Sr, Ba, Nd, and Ti and the striking negative Eu anomalies (Fig. 9) indicate fractionation of plagioclase, K-feldspar and Ti-Fe oxides. In comparison with S_1_, S_2_ and S_5_, the S_3_ and S_4_ granites show much more pronounced depletions in Ti, P, Sr and Ba, as well as extremely negative Eu anomalies (Fig. 10). Extensive magmatic fractionation is further supported by the ratios of Zr/Hf, Nd/Ta, Th/U and Rb/Sr. With the increase in the degree of magmatic fractionation, Zr/Hf, Nd/Ta, Th/U and ratios decrease, but the Rb/Sr ratio increases^50–52^. The Zr/Hf ratios of all five sections are 18.36–20.55 (S1), 20.14–20.18 (S2), 7.43–8.28 (S3), 8.90–11.53 (S4), and 25.92–38.24 (S5). The Nb/Ta ratios are 5.92–6.61, 5.52–5.54, 1.06–1.44, 0.82–2.58, and 7.73–7.74, respectively. The sections have Th/U ratios of 1.84–2.13, 1.94–2.04, 0.89–0.91, 0.91–1.16, and 3.97–4.30, respectively. The Rb/Sr ratios show much wider range: 7.99–10.24, 10.38–12.75, 60.13–108.81, 36.41–165.2, and 5.23–6.05, respectively. All the variations in the ratios from S_1_ to S_4_ suggest an increased degree of magmatic fractionation. It is worth noting that the S_5_ granite does not follow the increasing trend of magmatic evolution from S_1_ to S_4_. The S_5_ granite composition suggests that it experienced the lowest degree of fractionation.

Based on the primitive mantle-normalized diagrams (Fig. 9), the QGC can be classified into three groups: 1) S_1_ and S_2_ porphyritic biotite granites; 2) S_3_ and S_4_ equigranular biotite granites; and 3) S_5_ granite porphyry. Group 1 shows relatively flat REE patterns characterized by moderate enrichments in light REEs (LREEs) (with La/Y ratios ranging from 2.50 to 3.71) and slightly negative Eu anomalies (Eu/Eu^*^ = 0.144–0.249), suggesting that these samples have been weakly differentiated. In comparison, Group 2 has undergone strong fractional differentiation, as it exhibits flat REE patterns (with La/Y ratios ranging from 0.84 to 1.31) with much stronger Eu anomalies (Eu/Eu^*^ = 0.001–0.011). In contrast, Group 3 is characterized by strong enrichments in LREEs, with La/Y ratios ranging from 13.16 to 13.96 and the weakest Eu anomalies (with Eu/Eu^*^ ratios ranging from 0.31 to 0.222). On the primitive mantle-normalized spider diagrams (Fig. 9), both Group 1 and Group 3 show weak negative Ba, Sr, P, and Ti anomalies, whereas the anomalies of Group 2 are larger. All three groups record positive U and Th anomalies, but positive Ta anomalies occur only in Group 1 and Group 2.

Field observations indicate that a 0.4- to 1-m-wide baked margin is located at the contact between S_2_ and S_3_ and that NE-striking granite porphyry dikes (S_5_) cut through the other granites^26^, thus supporting our classification.. Additionally, positive Ce anomalies in zircon from S_2_ are strikingly higher than in zircon from S_2_, indicating that it was formed in a geochemical environment distinct from that of S_2_. Thus, S_1_ and S_2_ should be separated into individual groups. In summary, the QGC may be reasonably classified into four phases: P_1_, which contains S_1_; P_2_, which is composed of S_2_; P_3_, which comprises S_3_ and S_4_; and P_4_, which includes S_5_.

### Genetic relationship between plutonism and mineralization

Liu et al. (1997) obtained a garnet/pyroxene Sm–Nd age for the QGC of 160.8 ± 2.4 Ma, which was consistent (within error) with the Sm–Nd age for the massive-type skarn of 157 ± 6.2 Ma obtained by Lu et al. (2003)^25,39^. In contrast, according to the analyses of samples from massive-type skarn and vein-type greisen, Li et al. (2004) obtained a much younger Sm–Nd age of 149 ± 2 Ma, which matched the molybdenite Re-Os age of 151 ± 3.5 Ma obtained by Li et al. (1996) and a quartz fluid inclusion Ar–Ar age of 153.7 ± 0.9 Ma obtained by Wang et al. (2016)^28,30,31^. Furthermore, Yin et al. (2002) used muscovite ^40^Ar/^39^Ar dating to suggest that the timing of greisenization and its associated W–Sn–Mo–Bi mineralization ranged from 145 to 148 Ma^29^. Collectively, the age discrepancy is up to ~ 20 Myr.

Based on our field observations, the W–Sn–Mo–Bi mineralization of the Shizhuyuan deposit is intimately associated with greisenization; therefore, muscovite Ar–Ar dating is ideal candidate for assessing the timing of hydrothermal mineralization. According to our analytical results, greisen-type mineralization occurred ca. 154.2 ± 1.0 Ma. Within error, this age is consistent with molybdenite Re-Os dating (151 ± 3.5 Ma) conducted by Li et al. (1996)^31^. Wang et al. (2016) obtained a muscovite Ar–Ar age of 153.7 ± 0.9 Ma by carrying out geochronological Ar–Ar dating on a fluid inclusion in quartz and coexisting muscovite^30^. This age also agrees with our dates.

In conclusion, the QGC has two impacts on the Shizhuyuan deposit. (1) Heat supply: According to the thermal model of Mclaren et al. (1999)^5^, the heat derived from the high-heat-producing granites reaches a maximum ~ 10 Myr after its emplacement. Furthermore, the thermal disturbances caused by the high-heat-producing granites can drive hydrothermal fluid convection, resulting in mineralization. For instance, the mineralization in the Mount Elliott Cu-Au deposits, Australia, was produced by hydrothermal convection driven by the heat released from its associated high-heat-producing granite. The volume heat of the QGC estimated by the U, Th, and K contents is 5.89–14.30 μWm^−3^ (volume heat of high-heat-producing granite > 5 μWm^−3^), indicating their high heat production. Therefore, when mineralization occurred, the heat anomaly resulting from the QGC was quite strong (nearly its maximum strength), which promoted the development of hydrothermal convection around the QGC, leading to the generation of the Shizhuyuan deposit. (2) Metal supply: Field observations reveal that the Shizhuyuan W–Sn–Mo–Bi deposit is located at the endo-contact of the skarn and the porphyritic biotite granite (S_1_, S_2_) and equigranular granite (S_3_, S_4_), demonstrating their close spatial relationship^21,37^. In addition, as shown in Fig. 8a, the W contents in the S_1_, S_2_, S_3,_ and S_4_ granites are mostly 40–60 ppm; therefore, the QGC can supply sufficient metal for mineralization.

In summary, the QGC is temporally and spatially associated with the formation of the Shizhuyuan W–Sn–Mo–Bi deposit. Furthermore, the QGC provided heat and metals for these deposits.

## Conclusions

1. According to zircon LA-ICP-MS dating, the emplacement time of the Qianlishan granite complex is constrained to 155–151.7 Ma.
2. Based on petrological and geochemical characteristics, the Qianlishan granite complex can be classified into four phases: porphyritic biotite granites (Phase 1, Section 1); porphyritic biotite granites (Phase 2, Section 2); equigranular biotite granite (Phase 3, Sections 3 and 4); and granite porphyry dikes (Phase 4, Section 5).
3. The Qianlishan granite complex is temporally and spatially associated with the formation of the Shizhuyuan W–Sn–Mo–Bi deposit (mineralization time: 154 Ma).

## Appendix: Sampling and analytical methods—General remarks on sampling

### Granite samples

Five samples (Samples GL-13, 315–36, 490–21, 490–10, and 490–2) were collected from the QGC for dating analysis of accessory minerals (Figs. 2, 4; Table 6). Sixteen samples (Samples GL-12, GL-13, 315–35, 315–36, 490–21, 490–24, 490–9, 490–10, SZY-490-4a, SZY-490-4b, SZY-490-5a, SZY-490-5b, 490–2, 490–3, SZY-490-1a, and SZY-4.90-1b) were obtained for whole-rock geochemical analysis (Figs. 2, 4; Table 6).

**Table 6:** Lithology and location of samples.

Section	Sample	Lithology	Location
1	GL-13	Fine-grained porphyritic biotite granite	Taipingli Road, N 25°46′04″, E113°09′42″, H 272 m
	GL-12	Fine-grained porphyritic biotite granite	Taipingli Road, N 25°46′04″, E113°09′42″, H 272 m
2	315–36	Fine-grained porphyritic biotite granite	Main transport tunnel, Lever 500
	315–35	Fine-grained porphyritic biotite granite	Main transport tunnel, Lever 500
3	490–21	Coarse-grained equigranular grantie	Crossing between Tunnel C1 and P1, Level 490
	490–24	Coarse-grained equigranular grantie	Crossing between Tunnel C1 and P1, Level 490
4	490–10	Fne-grained equigranular granite	Crossing between Tunnel P4 and C6-C7, Level 490
	SZY-490-5a	Fine-grained equigranular granite	Crossing between Tunnel C6 and P4, Level 490
	SZY-490-5b	Fine-grained equigranular granite	Crossing between Tunnel C6 and P4, Level 490
	SZY-490-4a	Fine-grained equigranular granite	Main transport tunnel, Lever 350
	SZY-490-4b	Fine-grained equigranular granite	Main transport tunnel, Lever 350
5	490–2	Granitic porphyry	Main transport tunnel, Lever 490
	490–3	Granitic porphyry	Main transport tunnel, Lever 490
	SZY-490-1a	Granitic porphyry	Main transport tunnel, Lever 490
	SZY-490-1b	Granitic porphyry	Main transport tunnel, Lever 490
ore	YJW-8-B	Greisen	Main transport tunnel, Lever 350

Samples GL-12 and GL-13, which represent fine-grained porphyritic biotite granite (S_1_), were collected on the sides of Taipingli Road (25°46′04″ N, 113°09′42″ E). Samples 315–36 and 315–35 were collected from a microfine-grained porphyritic biotite granite (S_2_) in the main transport tunnel on Level 500. Samples 490–21 and 490–24 are medium- to coarse-grained equigranular biotite granites (S_3_) that were collected in Tunnel 490. Samples 490–9, 490–10, SZY-490-4a, SZY-490-4b, SZY-490-5a, and SZY-490-5b are fine-grained equigranular biotite granites (S_4_) that were collected in Tunnel 490. Samples 490–2, 490–3, SZY-490-1a and SZY-490-1b were collected from granite porphyry dikes (S_5_) in Tunnel 490.

In total, sixteen granite samples and one stockwork greisen were collected with a sledgehammer. Each sample weighed 5 to 10 kg. Samples GL-12 and GL-13 were fresh massive rocks sampled from the outcrops of granites beside Taipingli Road, and the weathered parts were chipped off using a hammer. All the other samples were fresh rocks collected with sledgehammers from granite outcrops in the tunnels.

### Stockwork greisen

Sample YJW-8-B, consisting of massive rock, was collected using a sledgehammer from stockwork greisen in the main transport tunnel, Level 350. It represents the greisen associated with W–Sn–Mo–Bi mineralization.

In summary, we conducted zircon LA-ICP-MS U–Pb dating and whole-rock major and trace element analyses on all five sections of the QGC. Muscovite Ar–Ar dating was carried out on the stockwork W–Sn–Mo–Bi to constrain the ore-forming age of the Shizhuyuan deposit.

### Thin-section preparation and optical petrography

Billets with a size of ~ 45 × 25 × 15 mm were cut from fresh field samples using a diamond blade. Then, they were planed and mounted on a 28 × 48 mm standard petrographic carrier glass using epoxy. After polishing with abrasive powders, the thin sections reached a thickness of 30 μm. To identify the mineralogies and textures of the rocks, polished thin sections were studied under a binocular microscope and an Olympus BX 51 polarizing microscope at China University of Geosciences (Beijing) (CUGB).

### Zircon LA-ICP-MS U–Pb dating

Five fresh samples weighing approximately 5 kg (Samples GL-13, 315–36, 490–21, 490–10, and 490–2) of the Qianlishan plutonic rocks were processed at the Central Laboratory of China Railway Resources Group. Zircon grains subjected to U–Pb dating were separated from these samples using conventional heavy liquid and magnetic techniques. Then, approximately 100 of the best-quality zircon grains from each sample were handpicked under a binocular microscope. These grains were mounted in epoxy and then polished. After the mounts were prepared, the grains were photographed using optical microscopy and cathodoluminescence (CL) imaging to reveal their internal morphologies, which were used to select grains and choose analytical spots. The CL images were obtained using a HITACHI S3000-N scanning electron microscope equipped with a Robinson backscattered-electron detector and a Gatan Chroma CL imaging system. These samples were analyzed using an Agilent 7900 quadrupole ICP-MS with a 193 nm coherent Ar-F laser and Resonetics S155 ablation cell in CODES (Centre for Ore Deposit and Earth Sciences) of Tasmania University. The NIST610 standard used for Pb correction was analyzed after every 15 unknowns. Th/U and Pb/Th mass bias downhole fractionation and instrument drift were corrected with the 91,500 zircon standard according to Wiedenbeck et al. (1995)^55^. Each zircon analysis comprised 30 s of blank gas measurements and 30 s of analysis time. The analyzed spots were 29 μm in size, and the laser was emitted at a frequency of 5 Hz with an energy density of approximately 2 J/cm^2^. Particles ablated by the laser were carried out by the flow of He carrier gas at a rate of 0.35 l/min into the chamber to be mixed with argon gas. Then, they were carried to the plasma torch. The Temora standard of Black et al. (2003) and the Plesovice standard of Sláma et al. (2008) were applied^56,57^. Data processing was carried out using concordia intercept ages on the Tera-Wasserburg plot utilizing ISOPLOT (Ludwig, v. 3.75, 2012, copyright@ BGC Berkeley Geochronology Center, 2006, available from: http://www.bgc.org/isoplot_etc/isoplot.html). The method of data reduction was described in Halpin et al. (2014)^58^. The estimation of random and systematic uncertainties followed the method in Paton et al. (2010)^59^.

### Whole-rock major and trace element analyses

Billets ~ 70 × 50 × 30 mm in size were cut from the selected fresh samples. They were cleaned with tap water and powdered to a grain size of < 200 mesh using an agate mill. Major and trace element analyses were carried out at the Central Laboratory of China Railway Resources Group, following the method of Liu et al. (2016)^60^. Major element concentrations were measured using a Thermo Fisher ARL Advant’X X-ray fluorescence (XRF) spectrometer, while LOI was measured using a CPA225D electronic analytical balance. The FeO contents of the samples were measured using the conventional wet chemical titration technique. Concentrations of trace elements, including REEs, were determined by ICP-MS (Thermo Fisher X-series 2) after full digestion. A detailed description of the test and the lower detection limit were documented in GB/T14506-2010^61^. The analytical errors ranged from 1 to 3% of the amount present. Additionally, the temperatures of the experiment ranged from 20–27 °C, and the humidity was 30% to 58%.

### Muscovite Ar–Ar dating

The stockwork W–Sn–Mo–Bi (Type 3) ore has the highest average ore grade and the largest amount of ore; it thus represents the main stage of mineralization and is an ideal candidate for studying the mineralization time of this deposit.

In this study, one fresh sample (~ 120 × 80 × 30 mm) was collected for muscovite dating from a greisen vein with mineralization (Type 3) at the contact with the skarn in Tunnel 450. As shown in Fig. 3, this sample contained mainly muscovite (~ 92%) and minor quartz (~ 5%) and K-feldspar (~ 2%). This muscovite sample was crushed to a size of 40–60 mesh, and muscovite separates were carefully handpicked to a purity of over 99% under a binocular microscope. After being washed in an ultrasonic bath using methanol and deionized water, the muscovite crystals were wrapped in aluminum foil and stacked in quartz vials. Then, they were irradiated for 1442 min in the B4 position of the swimming pool reactor at the Chinese Institute of Atomic Energy, Beijing. The Fangshan biotite standard (ZBH-25), which has an age of 132.7 ± 1.2 Ma and a potassium content of 7.6%, was used to monitor the neutron flux (2.65 × 10^13^ n cm^-2^ S^-1^). After the samples underwent cooling for approximately 100 days, step-heating ^40^Ar/^39^Ar analyses were conducted using an MM1200B mass spectrometer at the Ar–Ar laboratory, Institute of Geology, Chinese Academy of Geological Sciences, Beijing. The instrumental conditions and analytical methodology were described by^14^. Step-heating analyses were performed in a double-vacuum resistance furnace; at each temperature step, the muscovite crystals were heated for 10 min and then purified for 30 min. The Ca and K correction factors calculated based on the analyses of K_2_SO_4_ and CaF_2_ were (^36^Ar/^37^Ar_o_)_Ca_ = 0.0002389, (^40^Ar/^39^Ar)_K_ = 0.004782 and (^39^Ar/^37^Ar_o_)_Ca_ = 0.000806. A ^40 ^K decay constant of 5.543 × 10^−10^ year^−1^ was used in the age calculations^62^. ISOPLOT software (Ludwig, v. 3.75, 2012, copyright@ BGC Berkeley Geochronology Center, 2006, available from: http://www.bgc.org/isoplot_etc/isoplot.html) was used for data processing. The errors in the plateau ages are quoted at the 2σ level.
